# Patient-specific hiPSC-Podocytes as an in vitro model of genetic FSGS

**DOI:** 10.1038/s41598-025-25650-9

**Published:** 2025-10-28

**Authors:** Victoria Rose, Denise Fink, René Krüger, Annalena Kraus, Johannes Schödel, Mario Schiffer, Janina Müller-Deile

**Affiliations:** 1 Department of Nephrology, Uniklinikum Erlangen, Friedrich-Alexander-Universität Erlangen-Nürnberg, Ulmenweg 18, 91054 Erlangen, Germany; 2 Institute for Nanotechnology and Correlative Microscopy, INAM, Äußere Nürnberger Str. 62, 91301 Forchheim, Germany

**Keywords:** Cell biology, Molecular biology, Stem cells, Molecular medicine, Nephrology

## Abstract

Mutations in podocyte-specific genes are associated with genetic focal segmental glomerulosclerosis (FSGS), yet the potential for targeted treatments remains uncertain. Therefore, patient-specific models are essential for understanding cellular phenotypes, identifying personalized therapies, and avoiding ineffective treatments. This study utilized patient-specific human induced pluripotent stem cell (hiPSC)-Podocytes to investigate cellular phenotypic and functional alterations associated with genetic FSGS in vitro. HiPSC-Podocytes were generated from a patient with a mutation in the inverted formin 2 (*INF2*) gene, who showed a partial clinical response to steroid treatment. Compared to healthy donor-derived hiPSC-Podocytes, the patient-specific hiPSC-Podocytes exhibited decreased protrusion length, reduced levels of actin-associated markers, and alterations in INF2 protein levels. Additionally, actin filaments were disrupted, characterized by increased actin depolymerization. Next to the actin-modulating agent Bis-T-23, the steroid Solu-Decortin H (SDH) improved the actin cytoskeleton in the patient-specific cells, which aligned with the patient’s partial response to steroids. This underscores the importance of personalized treatment strategies based on specific cellular responses in genetic FSGS.

**Supplementary Information:**

The online version contains supplementary material available at 10.1038/s41598-025-25650-9.

## Introduction

FSGS is a renal lesion defined by the scarring of certain parts of some glomeruli, which are the kidney’s filtration units. A significant aspect is the effacement of podocytes, which are specialized cells forming the slit diaphragm of the filtration barrier. Recent studies indicate that genetic factors contribute to the disease, with genetic variants identified in up to 60% of children suffering from steroid-resistant nephrotic syndrome (SRNS)^1^ and in 30% of adults with FSGS^2^.

Variants in over 40 genes have been associated with SRNS^3^. Many of these genes are essential for the podocyte phenotype and function, including the formation of the podocyte slit diaphragm, the structure of the actin cytoskeleton, and the adhesion to the glomerular basement membrane^1,4^. Some genetic variants may disrupt normal podocyte function through indirect effects on the actin cytoskeleton, leading to podocyte foot process effacement^3,5–8^.

One common cause of autosomal dominant FSGS is heterozygous mutations in the *INF2* gene^6,9^. However, the impact of patient-specific single nucleotide polymorphisms has not been studied as extensively as cases with complete *INF2* knockout. Recent research in mice has demonstrated that knock-in models carrying INF2 mutations develop kidney disease spontaneously, indicating a direct pathogenic role of these mutations. In contrast, INF2 knockout mice do not typically develop kidney pathology on their own but exhibit kidney damage only when subjected to additional injury models. This suggests that the disease associated with INF2 mutations may result from a gain-of-function or dominant-negative effect of the mutant protein, leading to intrinsic cellular dysfunction. In contrast, complete loss of INF2 function requires an external insult to manifest kidney injury^10^. Additionally, the histological features of adult-onset genetic FSGS can vary significantly, even among individuals with the same genetic mutation^8^. This variability suggests that other factors, such as podocyte development, additional spontaneous genetic mutations, or environmental influences, may also shape the disease’s characteristics. These complexities pose challenges for traditional research methods like animal studies and cell culture models, which often fail to account for the patient’s genetic context. There is a pressing need for individualized disease models that reflect the patient’s unique genetic background to address these challenges. Therefore, we generated podocytes in vitro via the combination and modification of already existing methods for episomal reprogramming of dermal fibroblasts obtained from skin biopsies into human induced pluripotent stem cells (hiPSCs), followed by their differentiation into podocytes^11–13^. It has been demonstrated that these hiPSC-derived podocytes (hiPSC-Podocytes) more closely resemble natural podocyte morphology than human conditionally immortalized podocytes (ciPodocytes)^14^.

Here, the protocol was applied to a patient with genetic FSGS carrying a mutation in the *INF2* gene (c.217G > A; G73S), allowing for examining patient-specific podocytes in vitro. This included assessing diseased podocyte morphology, structure of the actin cytoskeleton, and expression of markers important for the podocyte phenotype. Furthermore, it facilitated testing of the patient-specific cellular response to potential therapeutic agents regarding their effects on the actin cytoskeleton and protrusion development. This personalized in vitro model aims to enhance our understanding of disease-specific alterations in podocytes, with the potential for more tailored therapeutic approaches.

## Materials and methods

### Generation of hiPSC-Podocytes derived from skin biopsies

A detailed step-by-step protocol for the generation of hiPSC-Podocytes was published previously^14^. HiPSC-Podocytes were derived from a patient and a healthy donor, both female and younger than 30 years old. The protocol was approved by the ethics committee of Friedrich-Alexander University Erlangen-Nuremberg (251_18B), and all probands and patients gave written informed consent. All experiments were performed according to the relevant guidelines and regulations. The quality of generated hiPSCs from the patient was analyzed for three individual clones. Seeding densities of nephron progenitor cells ranged from 8000 to 20,000 hiPSCs/cm^2^, depending on whether the specific experiment required single-cell analysis via imaging or more confluent cultures.

### Culture of human ciPodocytes

Human ciPodocytes (clone AB08) were cultured as previously described^15^.

### Differentiation in 3D

Embryoid body formation was performed at 10,000 hiPSCs/cm^2^ in a low-adhesive plate overnight on a shaking platform set to 60 rpm at 37 °C and 5% CO_2_. Differentiation into podocytes was performed using the same media as in 2D differentiation, with a media change achieved by allowing the spheres to sediment on agitated plates. 3D cultures were fixed with 4% PFA for 30 min, after washing with 1 × PBS.

### Sample preparation, SEM, and quantification

Ultrastructural analysis was performed via SEM. Samples were fixed with a combination of glutaraldehyde and formaldehyde. Fixation was performed as described earlier^16^.

### Chromosomal microarray analysis

Chromosomal stability of fibroblasts and hiPSCs was performed via the KaryoStat + ™ Genetic Stability Assay Service (#A52849, ThermoFisher Scientific).

### RNA isolation, cDNA synthesis, and quantitative PCR

500 ng of RNA was reverse transcribed into cDNA and diluted to 5 ng/µL. 1 µL was used for qPCR, and primer sequences used are listed in Table 1. The ∆∆Ct method was used for calculation, and *ACTB* served as a housekeeping gene.

**Table1:** Sequences of primers used for qPCR.

Gene	Forward sequence (5′-3′)	Reverse sequence (5′-3′)
*NANOG*	AGATGCCTCACACGGAGACT	TTGGGACTGGTGGAAGAATC
*OCT3/4*	CAGGGCCCCATTTTGGTACC	CTCAGTTTGAATGCATGGGAGAGC
*SOX2*	TTCACATGTCCCAGCACTACCAGA	ATGTGTGAGAGGGGCAGTGTGC
*REX1*	ACGTTTCGTGTGTCCCTTTC	TTAGGATGTGGGCTTTCAGG
*αSMA*	ACTGAGCGTGGCTATTCCTCCGTT	GCAGTGGCCATCTCATTTTCA
*SYNPO*	TAAGCAACCT…AACTGAGG	TTCTGGGCTAAAGCTAAC
*MAFB*	TTGTAACCAGAATCACCCTGAGGTC	CCAGGGTCAGGGATGGCTAA
*VIM*	AAATGGCTCGTCACCTTCGT	TTGCGCTCCTGAAAAACTGC
*WT1*	TCAGAGGCATTCAGGATGTG	TTATTGCAGCCTGGGTAAGC
*INF2*	CCCTAACCCTCAGCATGGCA	GCGCTTCCTCCTGGTGGTTC
*CTSL*	TAGAGGCACAGTGGACCAAG	CTCCATCCTTCTTCATTCATGCC
*GAPDH*	CAAGATCATCAGCAATGCCTCC	ATGATGTTCTGGAGAGCCCC
*HPRT*	GACCAGTCAACAGGGGACAT	AACACTTCGTGGGGTCCTTTTC
*ACTB*	ACCGAGCGTGGCTACAGCTTCACC	AGCACCCGTGGCCATCTCTTTCTCG

### Sanger sequencing

Primer flanking the *INF2* mutation (c.217G > A) (forward: CCCTAACCCTCAGCATGGCA, reverse: GCGCTTCCTCCTGGTGGTTC) were used to amplify the sequence via PCR. Products were separated on a horizontal 1% agarose gel at 120 V for 60 min, cut out, and extracted from the gel. Sanger sequencing was performed via the Mix2Seq sequencing service (Eurofins Genomics) with 75 ng of total DNA.

### Bulk RNA sequencing

Transcriptomic analysis of hiPSCs, nephron progenitor cells, and hiPSC-Podocytes from a healthy donor and a FSGS patient carrying the INF2 mutation, as well as from ciPodocytes, was performed by bulk RNA sequencing analysis performed by the transcriptome sequencing service of Novogene. Analysis was performed as previously described^17^. Podocyte-associated marker genes are based on published GSEA lists from the molecular signatures database (MSigDB *MENON_FETAL_KIDNEY_2_NEPHRON_PROGENITOR_CELLS, MENON_FETAL_KIDNEY_4_PODOCYTES, LAKE_ADULT_KIDNEY_ C2_PODOCYTES*) as well as from the original publication of the differentiation protocol^12,18,19^. Analysis of hiPSC, nephron progenitor cells and hiPSC-Podocytes was performed for two individual clones each and ciPodocytes in duplicates from the clone AB08.

### Immunocytochemistry

Cells were fixed in paraformaldehyde and incubated with 0.5% Triton-X-100, 1% bovine serum albumin, and 10% normal goat serum (1 h, RT). Primary antibodies were diluted according to Table 2 in 1 × PBS containing 1% bovine serum albumin and 3% normal goat serum and incubated overnight (4 °C). After washing 3 × with 1 × PBS for 5 min at RT, secondary antibodies were incubated for 1 h (RT). After washing, samples were mounted using Fluoromount-G containing DAPI.

**Table 2:** Antibody dilutions used for Immunocyto- and histochemistry and Western Blot Analysis.

Antibody	Company	Catalogue number	Immuno-cytochemistry	Immuno-histochemistry	Western blot analysis
Synaptopodin	Progen	61094	1:200	1:200	1:100
INF2 C-terminal	Proteintech	20466-1-AP	1:100	1:100	1:100
Podocin	Proteintech	20384-1-AP	1:200	1:100	–
Ki67	Abcam	ab15580	1:500	–	–
OCT3/4	Stem cell technologies	60093.1	1:100	–	–
SSEA4	Stem cell technologies	60062FI.1	1:100	–	–
Tra-1-81	Invitrogen	12-8883-82	1:100	–	–
Cortactin	Invitrogen	PA527134	1:200	–	1:300
Nephrin	Progen	GP-N2	–	1:100	
INF2 N-fragment	Sigma Aldrich	SAB1401801	–	–	1:100
RhoA	Santa Cruz	sc-418	–	–	1:25
Cathepsin L	Invitrogen,	BMS1032	–	–	1:500
GAPDH	Proteintech	10494-1-AP	–	–	1:5000
Phalloidin 647	Invitrogen	A22287	1:400	1:200	–
Alexa Fluor anti-mouse 488	Invitrogen	A11001	1:1000	1:200	–
Alexa Fluor anti-rabbit 555	Invitrogen	A31572	1:1000	1:200	–
Alexa Fluor guinea pig 488	Invitrogen	A11073	1:1000	1:200	–
Anti-mouse and HRP	Agilent technologies	P0447	–	–	1:10,000
Anti-rabbit HRP	Agilent technologies	P0448	–	–	1:10,000

### Immunohistochemistry

Samples were deparaffinized in xylol (3 × 5 min) and rehydrated in a descending ethanol series (5 min each: 2 × 100%, 2 × 96%, 1 × 80%, 1 × 70%, ddH_2_O). Samples were boiled in citric acid buffer for 15 min in a pressure cooker and rinsed in 1 × PBS. Staining was performed with the dilutions according to Table 2.

### Western blot analysis

30 µg of lysates was boiled in Laemmli buffer (70 °C for 10 min) and separated on SDS gels (8–12%) at 120 V for 1 h. Nitrocellulose membrane and 5% BSA were used and primary antibodies were diluted according to Table 2 in TBS and incubated overnight (4 °C). Membranes were washed 3 × with TBS-T and then incubated in secondary antibody for 1 h (RT).

### Classification and quantification of actin filaments

Total fluorescence of phalloidin was analyzed from confocal images using ImageJ. Classification of actin filaments was performed as previously described^20,21^. Type A represents actin bundles, type B actin filaments, and type C fragmented actin filaments. Evaluation was performed blinded. Quantification was performed on three individual experiments for all three clones, with more than 50 cells per replicate and more than 5 fields analyzed per well.

### Actin polymerization and depolymerization assay

Actin Polymerization Biochem Kit (#BK003, Cytoskeleton) with non-muscle actin (#APHL99, Cytoskeleton) and muscle actin (#APHL95), mixed 10:1, was used to analyze actin polymerization and depolymerization of control and patient hiPSC-Podocyte lysates (30 µg). Increase or decrease of fluorescence was measured at 395–440 nm over 60 min.

### Treatment of hiPSC-Podocytes

HiPSC-Podocytes were treated for 1 h with Bis-T-23 (30 µM) (Aberjona Laboratories, Inc., Beverly, MA), tacrolimus (10 µg/mL) (Prograf, Astellas Pharma), and SDH (1 µg/mL) (Jenapharm) in 150 µL basic medium per well and subsequently fixed and stained with phalloidin and DAPI. Quantification was performed in a black 96-well plate in triplicate for all three clones using a GlowMax plate reader and was normalized to DAPI signal.

### Statistical analysis

ImageJ was used for analyzing circularity, protrusion length and total fluorescence intensity. Dot plots were plotted as median plus range and statistical significance was calculated with GraphPad Prism using unpaired Welch’s t-test or one-way ANOVA. Significance (*p*-value) is indicated with asterisks.

## Results

### Patient with adult-onset genetic FSGS with an INF2 mutation

**Fig. 1:**
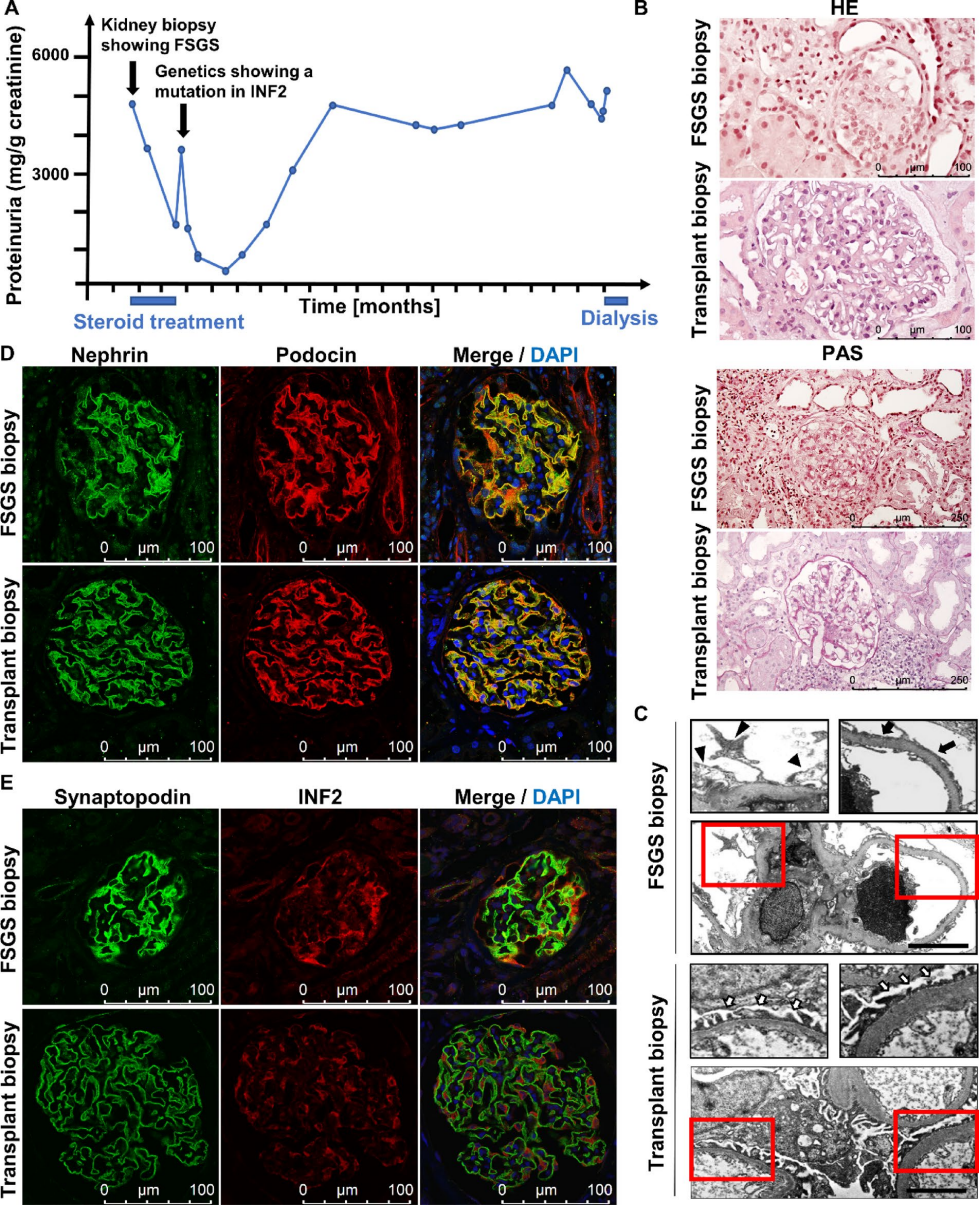
Characterization of the patient’s renal pathology. **(A)** Progression of proteinuria as mg/g creatinine over time (months) in the patient diagnosed with genetic FSGS. Timepoint of steroid treatment and dialysis is marked in blue on the x-axis. **(B)** Histological analysis of hematoxylin–eosin (HE) and periodic acid–Schiff (PAS) staining of a kidney biopsy from the FSGS patient compared to a biopsy of the transplanted kidney. Scale bars represent 100 µm for HE and 250 µm for PAS staining. **(C)** Transmission electron microscopy images from the FSGS biopsy, illustrating focally irregular, jagged podocyte morphology (black arrowheads) and segmental foot process effacement (black arrows). The healthy transplanted kidney biopsy serves as a control with regular podocyte foot processes (white arrows). Scale bars represent 10 µm. (**D–E**) Immunofluorescence staining of podocyte-specific and actin-associated markers. The slit diaphragm protein nephrin is shown in green and podocin in red **(D**). The actin-associated podocyte-specific marker synaptopodin is depicted in green, and INF2 protein in red **(E)**. Nuclei are in blue, stained with DAPI. Scale bars represent 100 µm.

We generated hiPSC-Podocytes from a patient diagnosed with juvenile-onset FSGS at the age of 17. At the time of diagnosis, this patient exhibited chronic kidney disease (CKD stage G4A3). After starting steroid treatment, the urine protein-to-creatinine ratio (UPC-ratio) improved from 5.113 mg/g creatinine to 1.803 mg/g creatinine (Fig. 1A). Histological examination of the renal biopsy revealed severe global glomerulosclerosis (29/35) and focal glomerulosclerosis (2/35). Additionally, there was 30–35% interstitial fibrosis and tubular atrophy (Fig. 1B). Electron microscopy showed irregular, jagged podocyte morphology and segmental foot process effacement (Fig. 1C). Genetic analysis identified a heterozygous autosomal dominant point mutation in exon 2 of the *INF2* gene, characterized by a nucleotide exchange from guanine to adenine at position 217 (c.217G > A), which results in an amino acid substitution at position 73 of the protein sequence, changing glycine to serine (G73S). Immunosuppressive therapy was discontinued after identification of the genetic cause of FSGS. However, proteinuria increased again, and the patient progressed to end-stage renal disease one and a half years later, ultimately receiving a living donor kidney transplant. The biopsy of the transplanted kidney shows normal podocyte foot processes and was used as a reference (Fig. 1B, C). Staining for common podocyte-associated marker proteins, such as synaptopodin, nephrin, and podocin, as well as the actin-associated protein INF2, displayed structural alterations and significant damage to the glomerular filtration barrier in the patient’s kidney biopsy compared to the healthy transplant biopsy (Fig. 1D, E).

### Quality control of patient- and healthy donor-derived hiPSCs regarding pluripotency and genomic stability

**Fig. 2:**
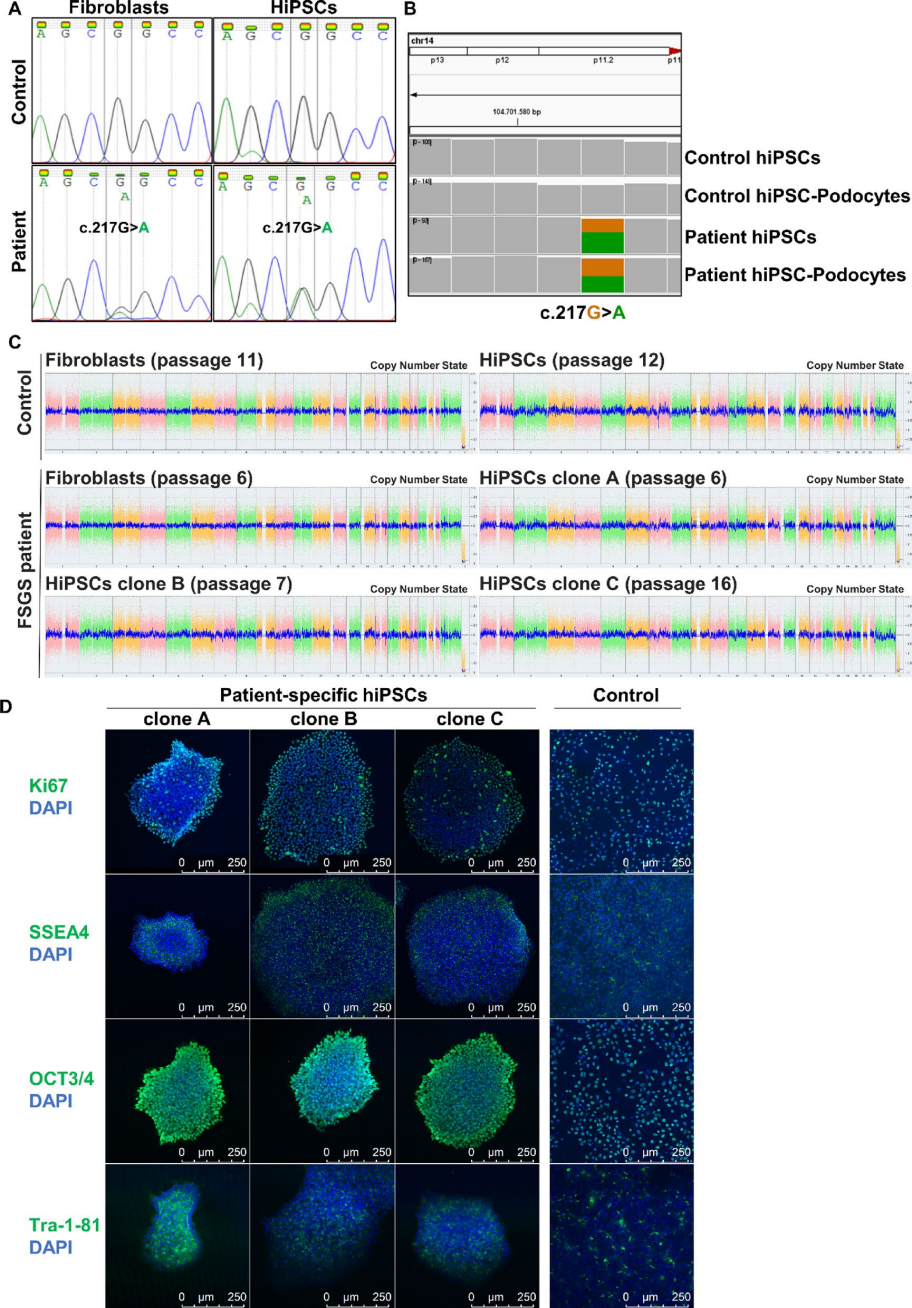
Genomic stability and pluripotency assessment of control- and patient-derived hiPSCs. **(A–B)** Sanger sequencing of fibroblasts and hiPSCs **(A),** alongside genomic data visualized using the Integrative Genomics Viewer for hiPSCs and terminally differentiated hiPSC-Podocytes **(B)**. These results illustrate the preservation of the patient’s genetic *INF2* variant (c.217G > A) throughout reprogramming and differentiation, with the variant absent in control cells. **(C)** Microarray analysis indicates no chromosomal aberrations before and after reprogramming in patient- and control-derived fibroblasts and hiPSCs. For patient-derived hiPSCs, three individual clones were analyzed. **(D)** Immunofluorescence staining for the proliferation marker Ki67 and pluripotency markers OCT3/4, SSEA4, and Tra-1-81 colocalized with nuclear staining in blue. All hiPSC clones showed equal expression of pluripotency markers. Scale bars represent 250 µm.

Dermal fibroblasts were cultured from skin biopsies derived from a patient with genetic FSGS and an INF2 mutation (c.217G < A; G73S) and a healthy donor. These fibroblasts were episomal reprogrammed into hiPSCs and subsequently differentiated into hiPSC-Podocytes following our recently published protocol^14^. To ensure high quality of the generated hiPSCs, we characterized both the patient-specific and healthy control hiPSCs in terms of genomic stability (Fig. 2A–C), proliferation capacity, and expression of pluripotency markers (Fig. 2D, E). The patient’s point mutation in the INF2 gene was preserved during reprogramming and differentiation, being present both in patient-specific fibroblasts and hiPSCs (Fig. 2A), as well as in the hiPSC-Podocytes (Fig. 2B). In contrast, the mutation was absent in all cells derived from the healthy control. Moreover, no chromosomal aberrations were detected before or after reprogramming in either cell line (Fig. 2C). The proliferation marker Ki67 and common pluripotency markers such as OCT3/4, SSEA4, and Tra-1-81 were expressed in all three individual clones of the patient-derived hiPSCs and in healthy control-derived hiPSC lines (Fig. 2D, E). This essential control of the generated cells shows high quality of the generated stem cells, and additionally, the transformation of the cell types during reprogramming and differentiation was also monitored (Fig. S1). We compared terminally differentiated hiPSC-Podocytes from a healthy donor to commonly used ciPodocytes to confirm podocyte identity (Fig. S2). Here, hiPSC-Podocytes expressed marker genes important for the podocyte phenotype and exhibited a more developed morphology, characterized by a branched network of secondary protrusions.

### Phenotypic differences of hiPSC-Podocytes derived from a patient with genetic FSGS

**Fig. 3:**
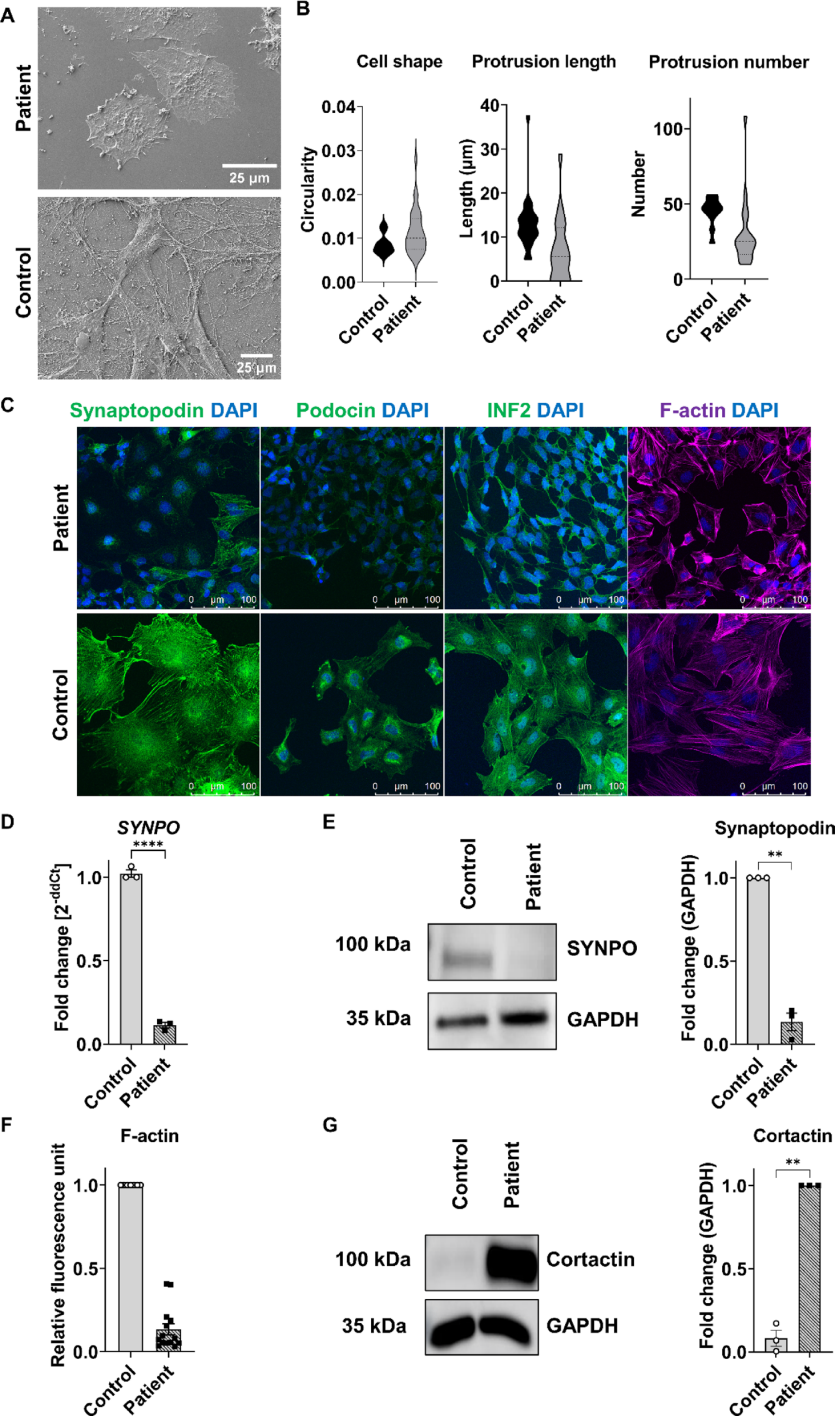
Phenotypic alterations of patient hiPSC-Podocytes. **(A)** Scanning electron microscopy images of control and patient hiPSC-Podocytes. Scale bars represent 25 µm. **(B)** Violin plots of quantified morphological features of individual cells (around 25 cells per cell line) from SEM imaging, including cell shape (circularity), protrusion length, and number. **(C)** Immunocytochemical staining of the podocyte-specific markers podocin and synaptopodin, the actin-associated protein INF2, as well as filamentous actin (F-actin) stained with phalloidin in control and patient hiPSC-Podocytes. Scale bars represent 100 µm. **(D–E)** Decreased synaptopodin expression in patient hiPSC-Podocytes, demonstrated at mRNA level via qPCR **(D)** and at protein level via western blot analysis normalized to GAPDH **(E)**. **(F)** Quantification of filamentous actin (F-actin) stained with phalloidin, expressed as relative fluorescence units (RFU). **(G)** Increased cortactin level in patient hiPSC-Podocytes, analyzed by western blot analysis and normalized to GAPDH. Statistical analysis was performed using an unpaired Welch’s t-test; * *p* < 0.05, ** *p* < 0.01, *** *p* < 0.001, **** *p* < 0.0001, n = 3.

Scanning electron microscopy demonstrated significant morphological differences in the patient-specific hiPSC-Podocytes compared to hiPSC-Podocytes derived from a healthy donor (control) (Fig. 3A). These cells exhibited a rounder cell body with shorter and fewer cellular protrusions, indicating reduced cell spreading (Fig. 3B). Staining for common podocyte markers further confirmed the altered podocyte phenotype, showing reduced levels of podocin and synaptopodin, as well as INF2, which is associated with the cytoskeleton and is responsible for the regulation of linear actin filaments (Fig. 3C). Inter-clonal differences were excluded by staining of key podocyte markers, such as podocin and synaptopodin (Fig. S3). Since typical podocyte markers were present but decreased in the patient-derived hiPSC-Podocytes in 2D culture, we also differentiated them in 3D to create conditions beneficial for the development and maintenance of slit diaphragm proteins. Here, the patient-specific 3D hiPSC-Podocyte cultures expressed podocyte markers similar to control 3D hiPSC-Podocyte cultures (Fig. S4). The substantial reduction in synaptopodin levels in 2D differentiated hiPSC-Podocytes was validated at both the mRNA (Fig. 3D) and protein (Fig. 3E) levels. Filamentous actin, stained with phalloidin, was altered in patient-specific cells with significantly lower levels of total phalloidin fluorescence (Fig. 3F). In contrast, the protein level of cortactin, which also promotes actin rearrangement, was increased compared to the control (Fig. 3G).

**Fig. 4:**
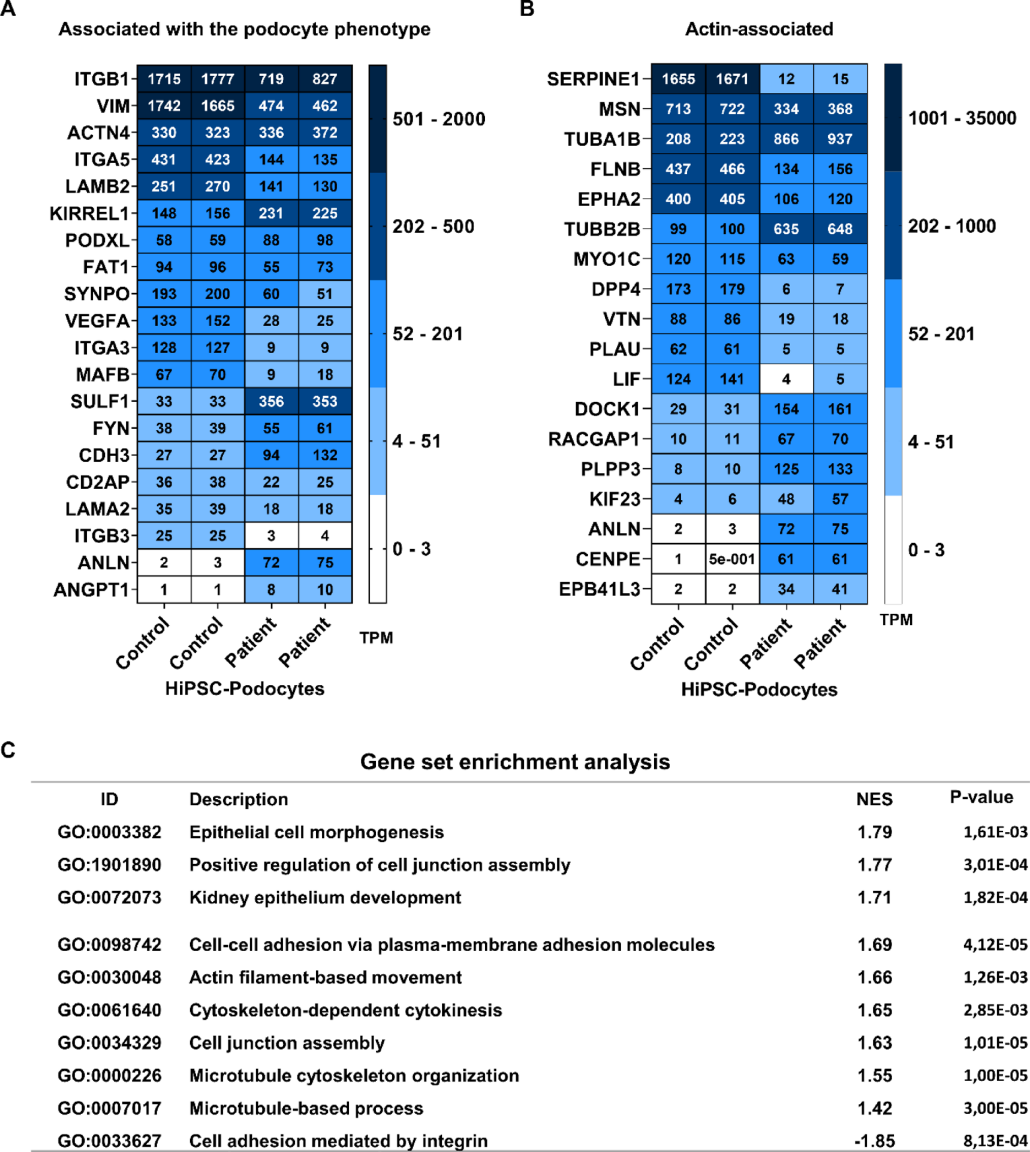
Comparative transcriptomic analysis of hiPSC-Podocytes from a FSGS patient and a healthy donor. **(A–B)** Transcriptomic differences were assessed for marker genes related to the podocyte phenotype **(A)** and to the actin cytoskeleton **(B)**. Two clones were analyzed for each cell line. Heatmaps with transcript per million (TPM) values from bulk RNA sequencing data. **(C)** Gene set enrichment analysis with normalized enrichment scores (NES) and *p*-values for differentially expressed genes. A positive score indicates higher activity in the patient hiPSC-Podocytes, while a negative score indicates higher activity of the pathway in control cells.

When analyzing large data from bulk RNA sequencing, we found that several markers important for maintaining the proper podocyte phenotype, such as *CD2AP*, *VIM*, *SYNPO*, *FAT1*, *MAFB*, along with adhesion via integrins (*ITGA3*, *ITGA5*, *ITGB3*) and extracellular matrix components (*LAMB2*, *VIM*) were expressed in both control and patient hiPSC-Podocytes, although many of these were found to be decreased in the patient-specific cell (Fig. 4A). Additionally, genes associated with actin-binding (*FLNB*, *MSN*, *MYO1C*) were also decreased, while genes related to microtubule formation (*TUBA1B*, *TUBB2B*, *KIF23*, *CENPE*), actin dynamics (*ANLN*), cytoskeletal protein-membrane anchoring (*EPB41L3*), and small GTPase activation (*DOCK1*, *RACGAP1*) were upregulated in patient hiPSC-Podocytes (Fig. 4B). Consistent with these findings, gene set enrichment analysis revealed that gene sets associated with epithelial development, cell–cell adhesion, actin cytoskeleton dynamics, microtubule cytoskeleton, and cell junctions were overrepresented in patient hiPSC-Podocytes compared to the control. In contrast, cell adhesion mediated by integrins was more active in control hiPSC-Podocytes (Fig. 4C).

### Altered INF2 dynamics and actin regulation in patient-specific hiPSC-Podocytes

**Fig. 5:**
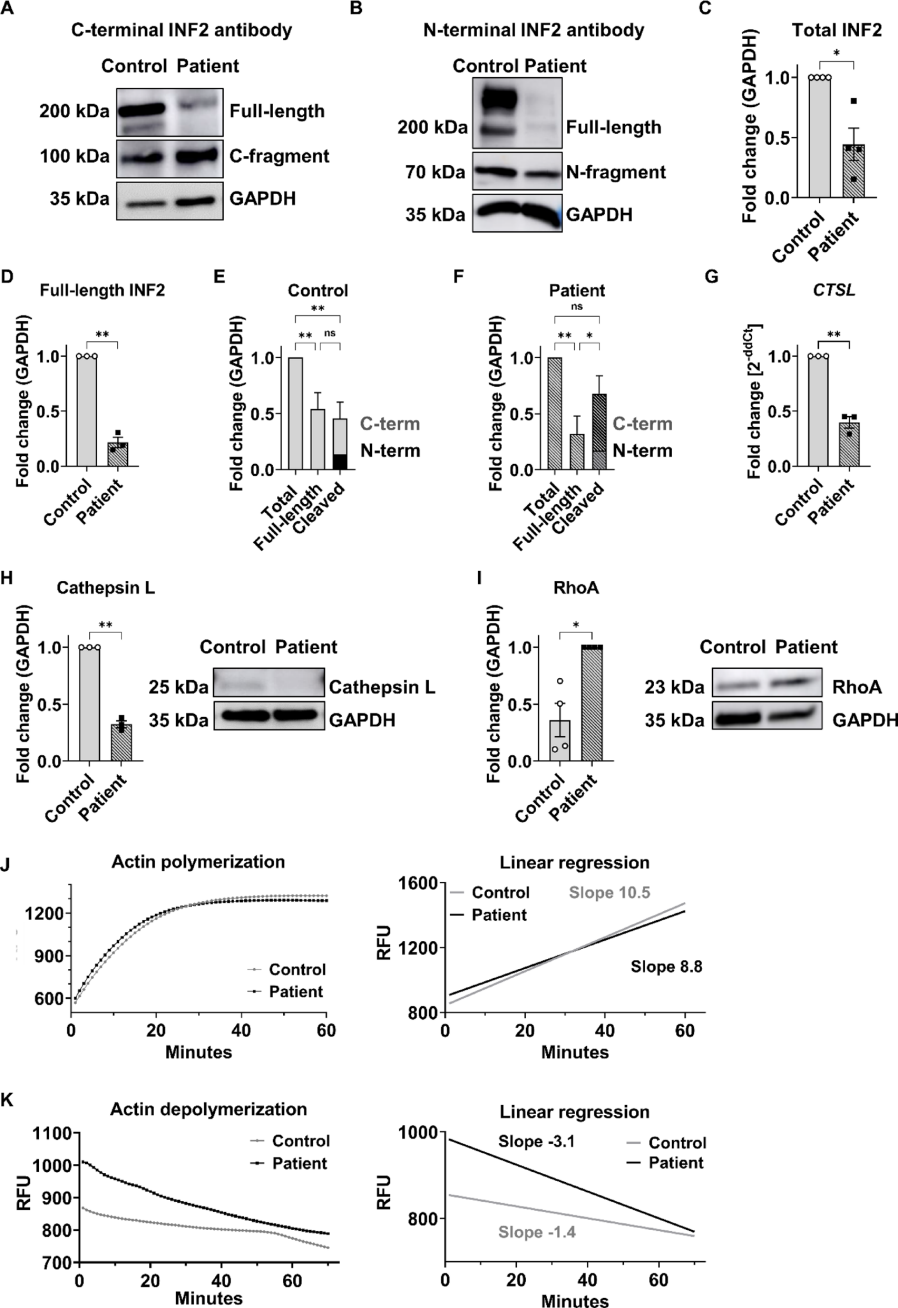
INF2 and actin dynamics in patient hiPSC-Podocytes. **(A–F)** Total and cleaved INF2 protein levels in patient hiPSC-Podocytes were compared to control using western blot analysis with antibodies targeting the C-terminus **(A)** and N-terminus **(B)** of INF2. The quantification of total INF2 protein levels (including both full-length and cleaved fragments) **(C).** Full-length protein was quantified in **(D)** and the distribution of uncleaved protein and cleaved C-terminal and N-terminal fragments relative to total INF2 levels is presented as a percentage for control **(E)** and patient **(F)** hiPSC-Podocytes. Data were normalized to GAPDH and analyzed using Welch’s t-test or one-way ANOVA. ns: not significant, * *p* < 0.5, ** *p* < 0.01, n = 3. **(G–I)** Cathepsin L expression was validated on mRNA level via qPCR with data normalized to *ACTB* **(G)** and confirmed decreased cathepsin L protein via western blot analysis, with normalization to GAPDH **(H)**. Welch’s t-test, ** *p* < 0.01. n = 3. **(I)** Protein expression of the small GTPase RhoA was examined, with corresponding quantification normalized to GAPDH. Welch’s t-test, * *p* < 0.5. n = 3. (**J**) Actin polymerization assay of protein lysates from control (grey) and patient (black) hiPSC-Podocytes with linear regression. The slope for control hiPSC-Podocytes is 10.5, while that for patient hiPSC-Podocytes is 8.8. This difference is not significant (n.s.). (**K**) Actin depolymerization assay of protein lysates from control (grey) and patient (black) hiPSC-Podocytes with linear regression. The slope for depolymerization in control hiPSC-Podocytes is − 1.4, whereas for patient hiPSC-Podocytes it is − 3.1, indicating a significant difference *p* < 0.0001. RFU: relative fluorescence units.

The formin INF2, which is involved in the polymerization and depolymerization of linear, unbranched actin filaments, can be cleaved into C- and N-terminal fragments by proteolytic cleavage at position 547 mediated by cathepsins^22–25^. Western blot analysis was performed using anti-INF2 antibodies that bind to either the N-terminal or C-terminal region of the INF2 protein. This showed a decrease in both total INF2 protein and full-length, uncleaved protein in patient hiPSC-Podocytes (Fig. 5A–D). The ratio of full-length INF2 protein to cleaved INF2 fragments remained consistent in control cells (Fig. 5E). In contrast, increased levels of INF2 fragments were detected in patient hiPSC-Podocytes, indicating a higher cleavage rate despite the low expression level of INF2 protein (Fig. 5F).

Cathepsins have been reported to be involved in the proteolytic cleavage of INF2 by proteolysis (Subramanian et al., 2020). Bulk RNA sequencing data revealed that several cathepsin isoforms, including L, B, D, Z, A, C, V, K, O, and H -predominantly cysteine proteases- were expressed in both patient and control hiPSC-Podocytes (Fig. S5). The downregulation of cathepsin L, which plays a crucial role in protein degradation and repair processes and is known to be important for podocytes, was confirmed at both mRNA (Fig. 5G) and protein levels (Fig. 5H). This indicates that the increased cleavage of the patient-specific INF2 protein in hiPSC-Podocytes is not directly linked to cathepsin L. The small GTPase RhoA plays a significant role in regulating actin dynamics and has previously been associated with the functionality of INF2. Western Blot analysis displayed a significant increase in RhoA levels in patient hiPSC-Podocytes, indicating a possible compensatory effect (Fig. 5I). Notably, the patient’s fibroblasts, which also carried the INF2 variant, exhibited no alterations in their cytoskeleton, INF2 expression, or cathepsin L expression (Fig. S6). These findings suggest that the observed effects are specific to the podocyte cell type in the patient carrying the INF2 mutation.

INF2 is known to facilitate both the polymerization and depolymerization of actin filaments. Its depolymerization activity is regulated by an autoinhibitory interaction between its N-terminal diaphanous inhibitory domain (DID) and C-terminal diaphanous autoregulatory domain (DAD) domains, whereas actin polymerization is unaffected by the DID-DAD interaction^25^. Interestingly, most genetic variants in the INF2 gene associated with genetic FSGS, including the variant identified in the featured FSGS patient, are located in the DID, which mediates INF2’s role in actin dynamics^9,26^. Therefore, we analyzed the ability of the patient’s hiPSC-Podocytes to polymerize and depolymerize actin filaments. While actin polymerization activity remained unaltered in the patient-specific hiPSC-Podocytes (Fig. 5J), actin depolymerization activity was significantly increased compared to the control (Fig. 5K).

### Exploring treatment responses in patient-specific hiPSC-Podocytes to immunosuppressive drugs and actin-targeting agents

**Fig. 6:**
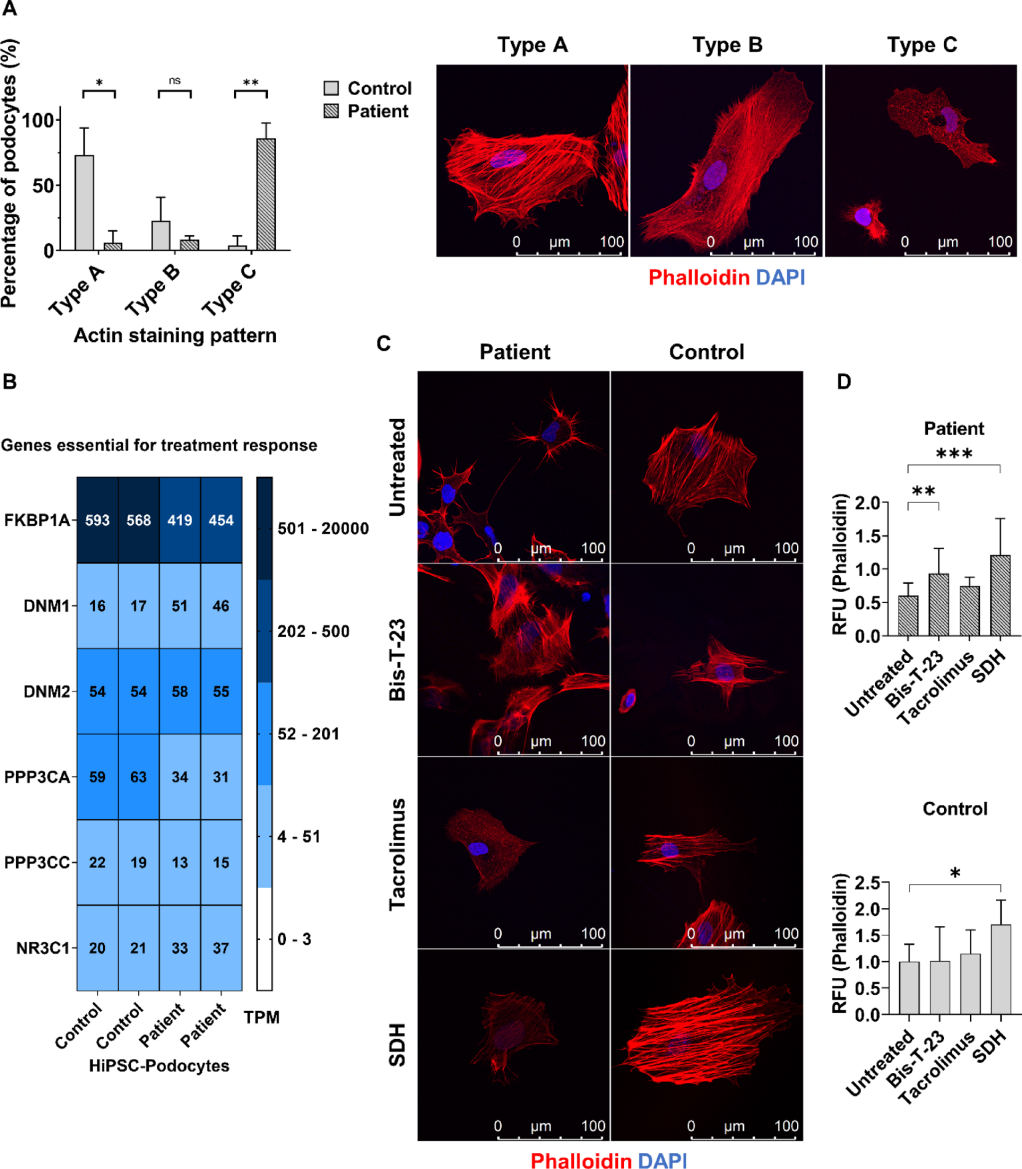
Altered actin cytoskeleton with improvements in the actin cytoskeleton of hiPSC-Podocytes. **(A)** Classification of the actin cytoskeleton in control and patient hiPSC-Podocytes after phalloidin staining (red), co-stained with DAPI (blue). Percentages are quantified based on the following classifications: Type A (more than 90% of the cell area showing bundled actin filaments), type B (unbundled actin filaments), and type C (fragmented actin filaments). Welch’s t-test, with significant differences indicated as * *p* < 0.05, ** *p* < 0.01, n = 3. Scale bars represent 100 µm. **(B)** Transcripts per million (TPM) from bulk RNA sequencing displaying the expression of genes essential for the response to treatment. **(C)** Representative images of untreated and treated hiPSC-Podocytes stained for F-actin with phalloidin in red and nuclei using DAPI in blue. Scale bars represent 100 µm. **(D)** Quantification of relative fluorescence units (RFU) of filamentous actin stained by phalloidin in control and patient hiPSC-Podocytes, both untreated and after 1 h of treatment with Bis-T-23 (30 µM), tacrolimus (10 µg/mL), and SDH (1 µg/mL). This analysis was performed in triplicate for each of the three clones using a plate reader. Values were normalized to the DAPI signal. One-way ANOVA, with significant differences indicated as * *p* < 0.05, ** *p* < 0.01, **** *p* < 0.0001, n = 3.

The actin cytoskeleton of the podocytes was analyzed using a scoring system previously published for the evaluation of filamentous actin^20,21^. Three types of actin filaments were classified: Type A, which features thick actin bundles covering more than 90% of the cytosol; Type B, characterized by thin and unbundled actin filaments; and Type C, which describes a disrupted cytoskeleton with only fragmented, short filaments. Notably, the patient hiPSC-Podocytes exhibited only type B and C filaments, while the control cells primarily demonstrated type A and some type B (Fig. 6A). The disrupted actin filaments in the patient hiPSC-Podocytes align with the altered podocyte phenotype and the accelerated actin depolymerization rate.

Genetic FSGS has limited treatment options and usually does not respond to immunosuppressive therapy. However, prior to genetic diagnosis, the patient did respond to steroid treatment with a partial remission of proteinuria (Fig. 1A). To analyze the cellular response to different immunosuppressants commonly used in clinical practice, as well as actin-reacting agents, we treated hiPSC-Podocytes derived from the patient and a healthy control with tacrolimus, SDH, and the actin stabilizing agent Bis-T-23. Bis-T-23 enhances the natural oligomerization of dynamin, promoting actin polymerization^27,28^.

In both patient and control hiPSC-Podocytes, genes related to pathways essential for treatment response were expressed (Fig. 6B). Transcriptomic levels of *DNM1* and *DNM2* encoding dynamin, which is the primary target of Bis-T-23, *FKBP1A* encoding FK506 binding protein, which is the binding partner of the calcineurin inhibitor tacrolimus, *PPP3CA* and *PPP3CC* encoding the calcineurin isoforms and *NR3C1* encoding the glucocorticoid receptor were all expressed, suggesting potential for direct effects of steroids and Bis-T-23 on podocytes.

After treatment, we quantified changes in fluorescence intensity of phalloidin, which stains filamentous actin (Fig. 6C, D). Following SDH treatment, we observed a significant increase in total F-actin fluorescence in both control and patient hiPSC-Podocytes. Moreover, treatment with Bis-T-23 resulted in an improvement of the filamentous actin cytoskeleton in patient hiPSC-Podocytes. In contrast, tacrolimus did not significantly alter F-actin levels in either group. To ensure equal cell numbers during treatment, DAPI signal alone was analyzed and showed no significant difference in the samples (Fig. S7).

In summary, we successfully generated podocytes from a patient with genetic FSGS in vitro, preserving the patient’s genetic background and allowing us to study the disease phenotype individually. We hypothesize that some patients with genetic FSGS may benefit from immunosuppressive treatments due to their direct effects on podocytes. This model enables us to analyze patient-specific podocytes ex vivo in-depth and estimate the potential effects in a personalized manner.

## Discussion

Adult-onset genetic FSGS is typically inherited as an autosomal dominant disease with variable penetrance^29^. Patients with genetic FSGS often resist immunosuppression, although some may still benefit from specific therapies^30–32^. However, the considerable toxicity associated with these therapies complicates empirical treatments for genetic FSGS, especially in the absence of evidence for their efficacy. Genetic variants may increase the vulnerability of podocytes to stress, potentially requiring modifier genes or additional metabolic, hypertensive, or other stressors for full development of the diseased phenotype^29^. For instance, genetic *ACTN4* variants contribute to cytoskeletal disorganization, impairing podocyte resistance to mechanical stress^7^.

Patient-specific ex vivo podocyte models are urgently needed to better understand the diseased podocyte phenotype and investigate individual responses of podocytes to treatments. This is crucial for identifying patients who may benefit from targeted therapies. Variants in the *INF2* gene have been studied in various model organisms, including zebrafish^33^, mice^10,34^, and drosophila^35^, as well as in vitro using human primary urinary epithelial cells^35^, ciPodocytes^36^, HEK293 and HeLa cells^37,38^. In these studies, knockdown of INF2 was mediated by morpholinos, siRNA, or CRISPR/Cas9 technology to investigate the role of INF2^33,39^. However, caution is necessary because ciPodocytes are known to dedifferentiate in vitro and behave differently than podocytes in vivo^40^. Additionally, the transfection of siRNA and injection of morpholinos can lead to inconsistent knockdown efficiency, and CRISPR/Cas9 may induce unintended off-target effects^40^. These models also do not fully represent the genetic background of the individual patient.

To address this, we derived patient-specific podocytes from a patient with genetic FSGS using our previously published protocol^14^. We cultured dermal fibroblasts from a skin biopsy of the patient, who carries a mutation in the *INF2* gene (c.217G > A; G73S), reprogrammed them into hiPSCs, and subsequently differentiated them into podocytes that maintained the patient’s genetic background. This enabled detailed characterization of patient-specific phenotypic and functional alterations and facilitated investigation of the individual response of these podocytes to treatment. Considering the entire genetic background of the patient, this may enhance the potential for clinical translation. The diseased patient-specific hiPSC-Podocytes were compared to those generated from a healthy donor to identify any pathological changes. The control was age- and sex-matched to characterize phenotypical and functional alterations in podocyte biology related to FSGS. The generated hiPSC-Podocytes from both healthy controls and a patient carrying the INF2 variant (c.217G > A; G73S) displayed characteristic podocyte markers and morphological features, providing a robust platform for comparative analyses. While our hiPSC-Podocytes represent an improved model and personalized in vitro model over traditional podocyte cell lines, they have inherent limitations. Two-dimensional cultures do not fully recapitulate the complex in vivo microenvironment, particularly the interactions with other glomerular cell types. Future advancements involving three-dimensional glomerular models, such as spheroids and kidney organoids, are anticipated to provide more physiologically relevant conditions that support the development of complex podocyte architecture, such as interdigitating processes.

To ensure high-quality and reproducible hiPSC-Podocytes, we implemented rigorous quality control measures. Comparative analyses of fibroblasts and hiPSC clones—before and after episomal reprogramming- focused on karyotypic stability and retention of the patient-specific INF2 mutation. We also evaluated hiPSC morphology, growth behavior, and pluripotency marker expression. To maintain consistency, we established working cell banks of well-characterized hiPSC clones for subsequent differentiation.

In this study, three individual hiPSC clones derived from the patient were differentiated into hiPSC-Podocytes. Differentiation efficiency was monitored via bulk RNA sequencing, immunofluorescence staining and qPCR and similar marker expression patterns were observed in all clones, suggesting minimal intra-clonal variability (Fig. S3).

However, with this approach, the findings cannot be exclusively related to the specific INF2 gene variant as the sole cause of the findings. To isolate the impact of the genetic cause on the diseased phenotype, an isogenic control, generated by reversing the patient’s single-nucleotide polymorphism in the patient-specific hiPSC line using CRISPR/Cas or introducing this mutation in a control hiPSC line, with subsequent functional studies, would be necessary to deepen the understanding of the relationship between gene mutations and disease pathogenesis.

The work presented here was limited to hiPSC-Podocytes derived from a single patient with genetic FSGS with an INF2 mutation in the DID as a proof of concept. Expanding this study to include a larger cohort of patients with different FSGS-related mutations would be desirable to increase the translational value of this model in the future. Comparative analyses across multiple mutations will help to identify potential common pathogenic mechanisms as well as individual differences in the disease presentation. Interestingly, podocytes harboring a patient-specific S186P INF2 mutation showed alterations in cell spreading and the actin cytoskeleton, which have been attributed to an INF2 gain-of-function effect^41^. However, some findings appear contradictory—for example, decreased cortactin levels in podocytes with S186P mutation, whereas in the here described podocytes with G73S mutation, cortactin expression was increased. These discrepancies may stem from differences in the mutation sites (G73S and S186P), as well as in the different experimental setups. In our study, podocytes potentially compensated for cytoskeletal disruption by upregulating cortactin and microtubule components, while in the previous study, podocytes were grown out of organoids and seeded onto micropatterned surfaces, possibly inducing additional stress. Furthermore, there is limited clinical information about the disease history of the patient carrying the S186P mutation, which hampers correlations between INF2 mutations and disease progression. It is also important to note that the precise role of INF2 in FGSG remains incompletely understood, and the key pathogenic pathways are still being elucidated. For instance, INF2 knock-out mice do not develop FSGS^25^, whereas INF2 R218Q knock-in mice only show disease after additional injury with puromycin aminonucleoside (PAN) treatment^10^. Similarly, patient-derived kidney organoids with the S186P INF2 mutation displayed nephrin pattern alterations even without a second hit. Collectively, these findings highlight the necessity of employing models that encompass the patient’s entire genetic background, as done in our study, rather than focusing solely on the specific INF2 mutation. Future studies are necessary to investigate multiple INF2 mutations within the DID using human models to better understand their pathogenic roles and improve disease modelling in vitro.

The INF2 protein consists of multiple domains: The N-terminal DID, dimerization domain (DD), formin homology (FH) 1 and FH2 domains, and the C-terminal DAD^22,42,43^. The FH2 domain accelerates actin filament assembly, while the C-terminal region promotes depolymerization^44^. DID binds to DAD of other formins, such as mDia, and inhibits depolymerization. In contrast, this interaction does not affect INF2-mediated polymerization^22,45^. Here, patient hiPSC-Podocytes exhibited predominantly fragmented actin and increased actin depolymerization. This indicates that the patient-specific *INF2* mutation disrupts autoinhibition.

The small GTPases RhoA, Rac1, and Cdc42 have been reported to modulate INF2 function^46^. While wildtype INF2 likely functions through direct competition with mDia protein rather than by activation via RhoA, many disease-causing INF2 mutants appear to increase interactions between INF2-active Cdc42 in HEK293 cells^26,37^. Increased RhoA protein levels were found in patient hiPSC-Podocytes, indicating that actin reorganization may be modulated by this GTPase in conjunction with the *INF2* mutation. Additionally, cortactin levels were elevated, likely as another compensatory response for cytoskeletal stabilization^47^.

Proteolytic cleavage of INF2 by cathepsins occurs at position 547, resulting in the accumulation of the N-terminal DID fragment at the cell edges, where it interacts with mDIA formins^25^. Consistent with previous studies, the patient’s *INF2* mutation did not impede cleavage of the full-length INF2 protein^25^. However, lower levels of total INF2 protein were found. Here, more INF2 fragments were found than the uncleaved INF2 protein in patient hiPSC-Podocytes. Most cathepsins, including cathepsin L, were downregulated, suggesting that the imbalance between full-length INF2 protein and INF2 fragments could arise from decreased stability or cleavage by other proteases. This downregulation of cathepsin L and INF2 levels and altered actin cytoskeleton were not observed in patient-specific fibroblasts, despite an identical genetic background. This indicates that the impact of the patient-specific INF2 mutation only affects the specialized podocyte phenotype. Future studies might include comparative analyses of actin-regulatory protein networks and assessment of podocyte-specific stress response pathways to elucidate mechanisms underlying this cell type-specific vulnerability.

While some immunosuppressive agents can impact the cytoskeleton independently of immune modulation, the clinical response of certain genetic variants of FSGS remains largely trial-and-error^30,48,49^. Most patients with genetic FSGS are steroid-resistant, but direct effects of steroids on podocytes have been described^50^. Furthermore, dexamethasone treatment protected podocytes from PAN injury by inhibiting actin filament disruption and apoptosis^51,52^. Carriers of WT1 variants were more likely to respond to calcineurin inhibitors (CNI) compared to carriers of other mutations. However, decisions regarding the continuation of CNI treatment in responsive monogenic SRNS should be made on a case-by-case basis^32,53^. Bis-T-23 has been shown to increase actin polymerization in injured podocytes in diverse kidney disease models^28,49^. Here, Bis-T-23 increased F-actin levels in the patient-specific hiPSC-Podocytes carrying the INF2 mutation. SDH treatment helped stabilize the cytoskeleton, paralleling the patient’s positive response to steroid treatment in the clinic. Dynamin and glucocorticoid receptors were expressed in patient hiPSC-Podocytes, allowing for direct effects of Bis-T-23 and SDH on these cells. Unlike what has been described for patients with WT1 mutation, tacrolimus did not remodel the control or patient hiPSC-Podocytes^54^. This underscores the importance of patient-specific in vitro models in understanding individual alterations and both short-term and long-term treatment responses at the cellular level. Linking in vitro findings with detailed clinical phenotypes is crucial to advance personalized medicine. Including basic clinical data of the index patient highlights the importance of integrating more detailed longitudinal patient data in future studies. Future studies should extend the treatment duration to assess sustained efficacy, possible toxicity, and off-target effects. This will be particularly important to bridge the gap between in vitro findings and clinical applicability.

In summary, patient-specific hiPSC-Podocytes offer multiple advantages for investigating genetic FSGS in vitro. Here, the patients’ diseased cells can be analyzed without gene manipulation methods like CRISPR/Cas or siRNAs, which carry risks of off-target effects and do not fully represent the patient’s genetic background.

## Supplementary Information

Below is the link to the electronic supplementary material.

Supplementary Material 1

Supplementary Material 2

## Data Availability

The bulk RNA sequencing data supporting the findings of this study are available in the NCBI Gene Expression Omnibus database under the accession number GSE254563. Raw data are not available due to patient privacy concerns. All other data are available in the published text or the supplementary materials.
