# Supplementary figures and images for: Patient-specific hiPSC-Podocytes as an in vitro model of genetic FSGS
Source: Sci Rep. 2025 Oct 28;15:37730. doi: 10.1038/s41598-025-25650-9 (PMC12569161; doi:10.1038/s41598-025-25650-9)

Blots cropped: Figure 3

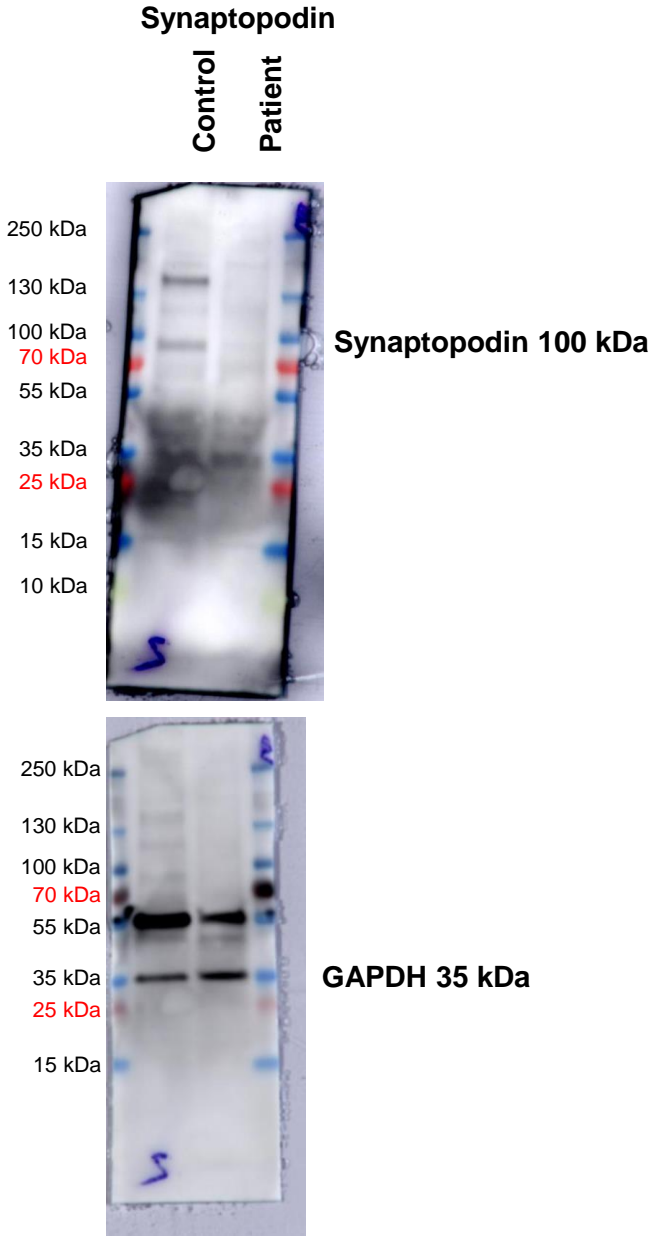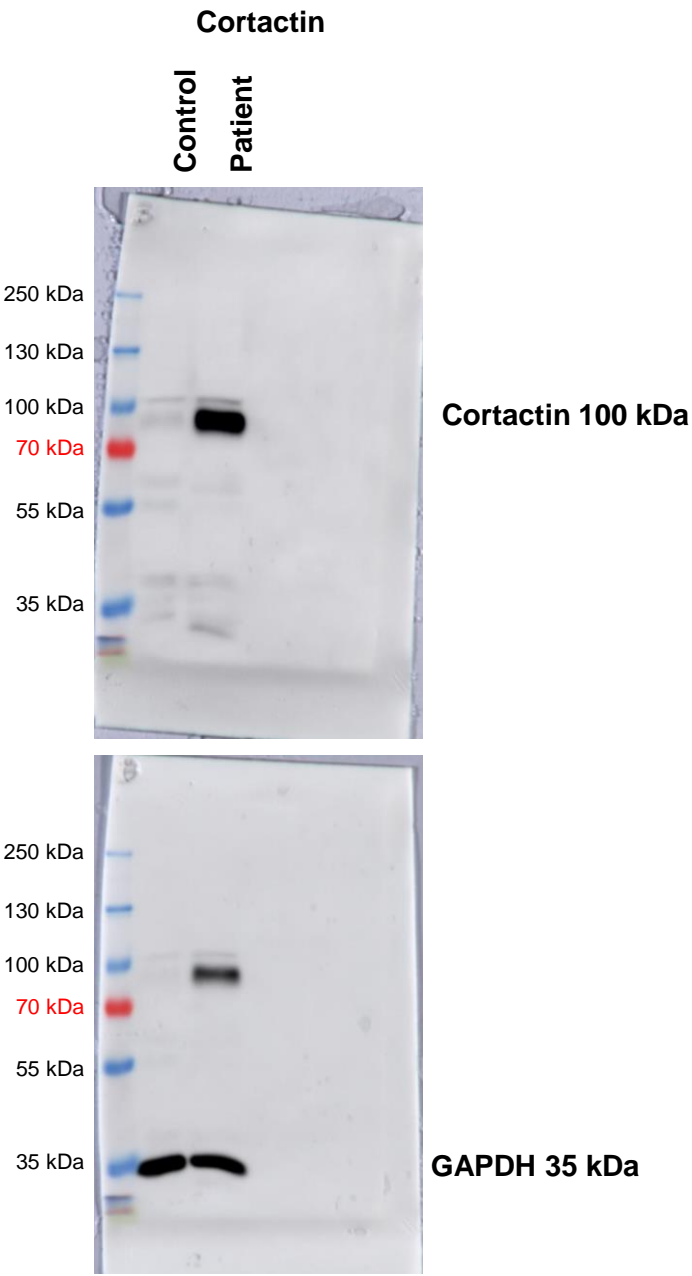

Blots uncropped: Figure 5

N-terminal INF2

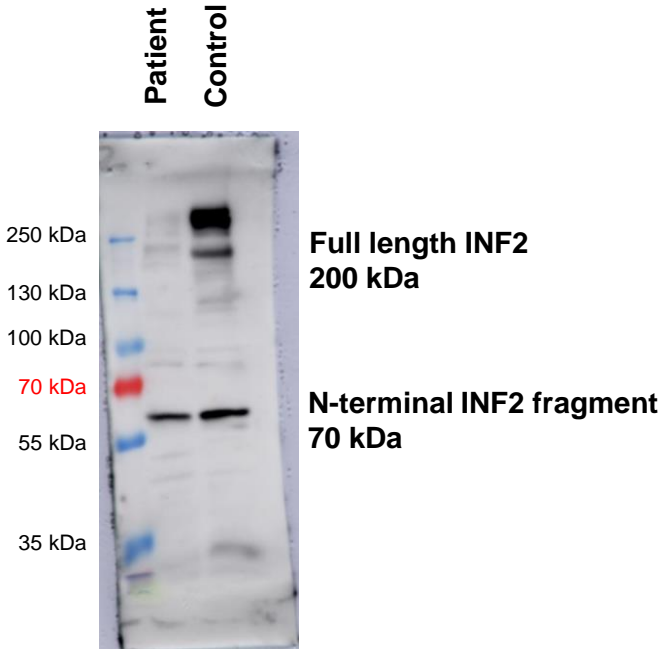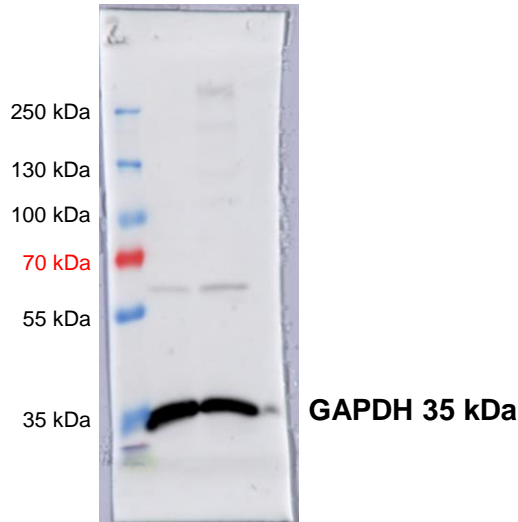

C-terminal INF2

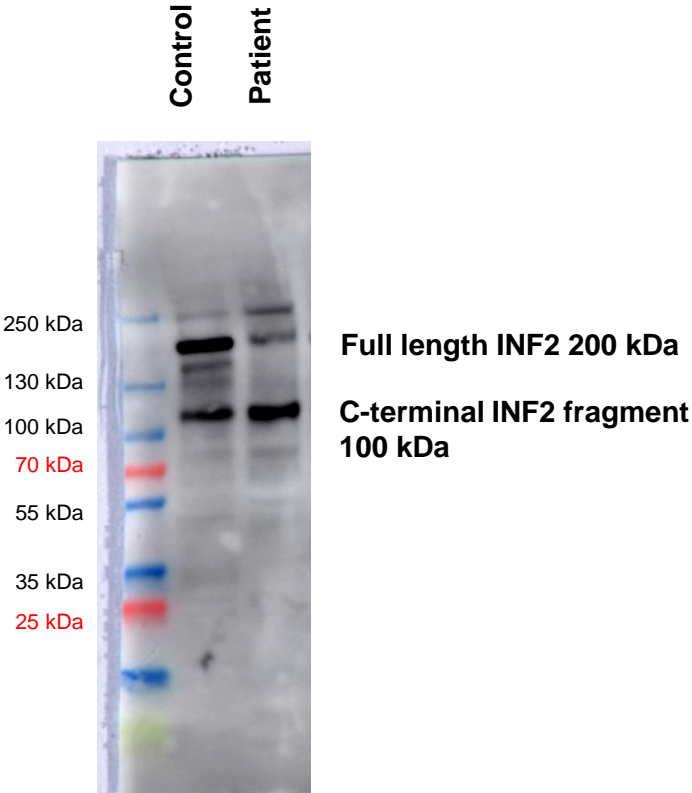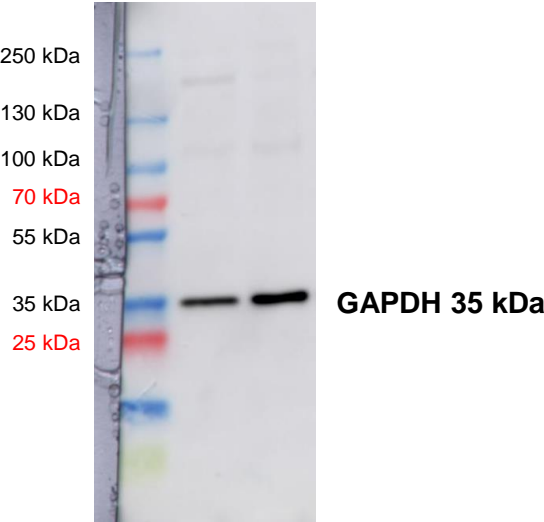

Cathepsin L

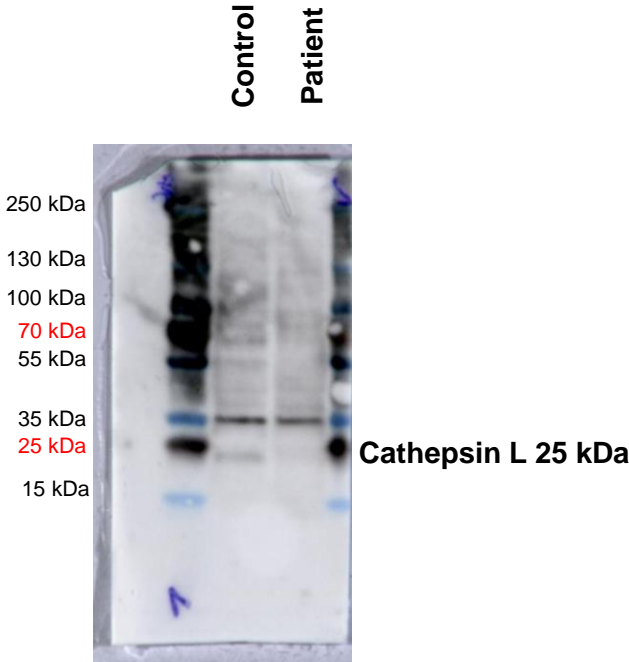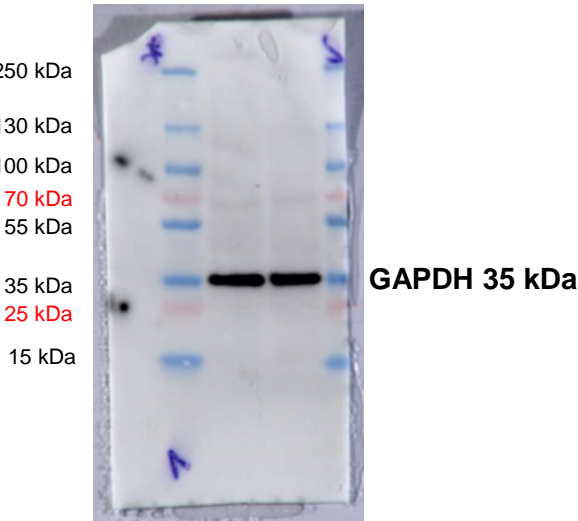

RhoA

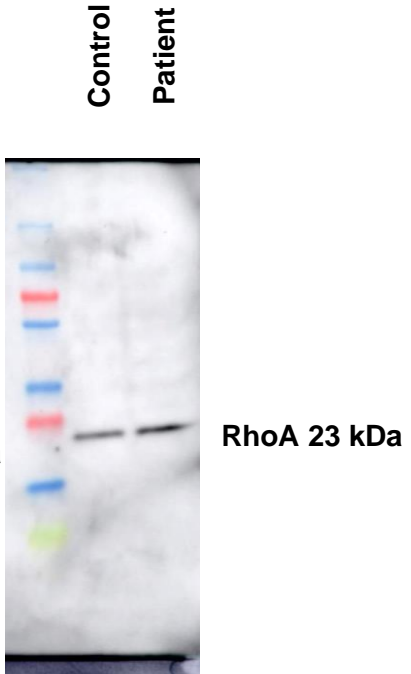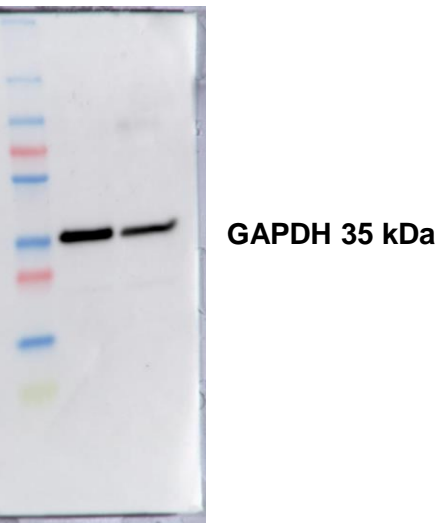

Supplement: Supplementary file 1 — Supplementary Material 1 [file 41598_2025_25650_MOESM1_ESM.pdf]
