# Supplementary material for: Patient-specific hiPSC-Podocytes as an in vitro model of genetic FSGS
Source: Sci Rep. 2025 Oct 28;15:37730. doi: 10.1038/s41598-025-25650-9 (PMC12569161; doi:10.1038/s41598-025-25650-9)
Supplement: Supplementary file 2 — Supplementary Material 2 [file 41598_2025_25650_MOESM2_ESM.docx]

**Supplemental designation**

**Figure S1** provides data for the validation of the protocol for the generation of healthy control hiPSC-Podocytes by tracking cell transformation during reprogramming and differentiation. **Figure S2** compares the generated hiPSC-Podocytes derived from a healthy control donor with ciPodocytes regarding podocyte-associated marker expression and morphology. **Figure S3** displays the differentiation efficiency of all three individual patient hiPSC clones by immunofluorescence staining of podocin and synaptopodin, as well as changes in transcriptomic expression of common pluripotency- and podocyte-related marker genes during differentiation. **Figure S4** shows patient-specific and control hiPSC-Podocytes cultured in 3D with expression of podocyte markers but with structural alterations in the patient-specific spheres. **Figure S5** displays transcriptomic expression of cathepsin isoforms in control and patient hiPSC-Podocytes from bulk RNA sequencing data. **Figure S6** demonstrates unaltered actin filaments, INF2 and cathepsin L protein expression in control- and patient-derived dermal fibroblasts. **Figure S7** displays DAPI signal of control and patient hiPSC-Podocytes before and after treatment, with no significant changes in cell number.

**
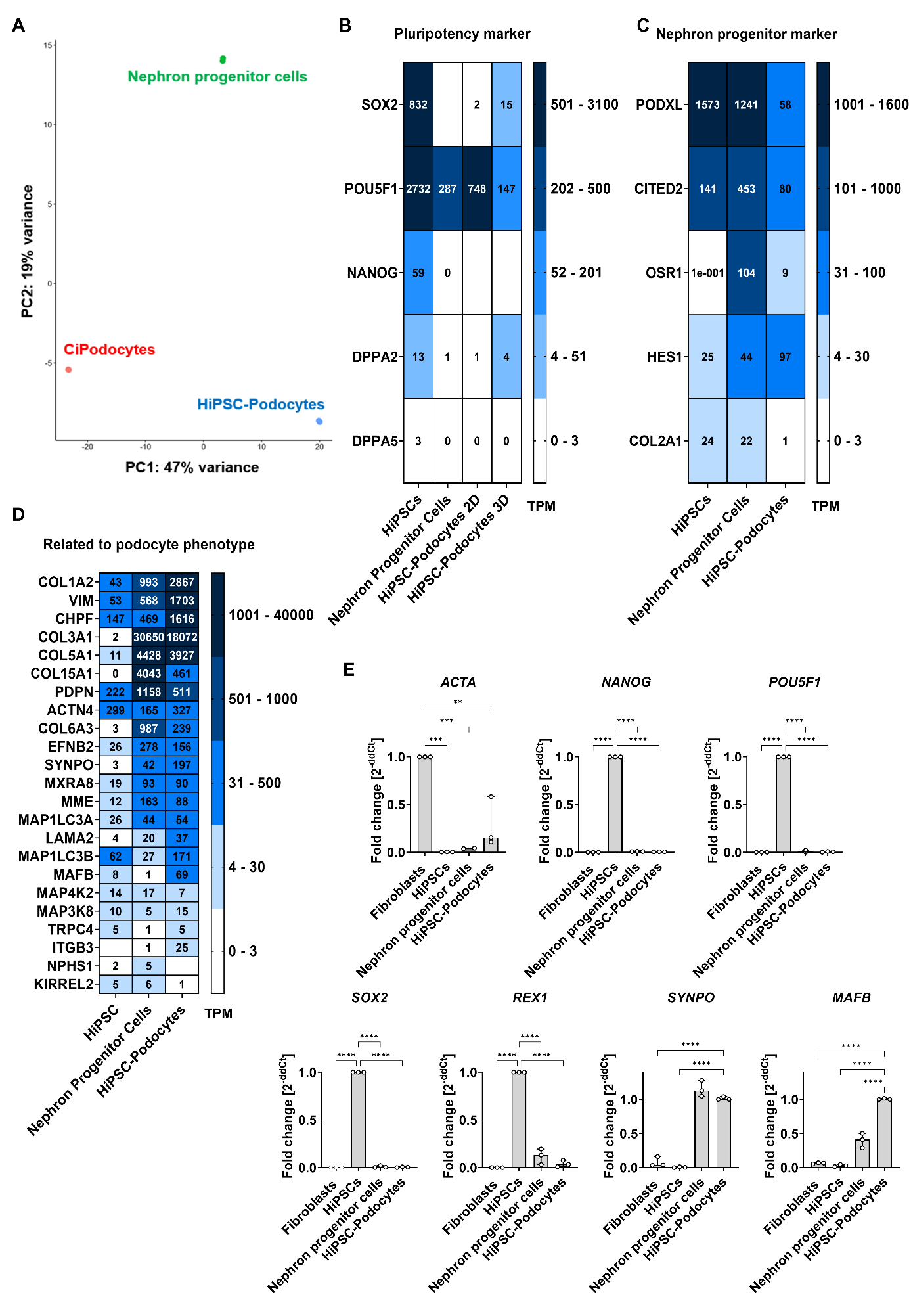
**

Figure S1. **Validation of the protocol for the generation of healthy control hiPSC-Podocytes by tracking cell transformation during reprogramming and differentiation. (A)** The relation among nephron progenitor cells, hiPSC-Podocytes and ciPodocytes is visualized by principal component (PC) analysis of bulk RNA sequencing data. Two different clones were analyzed for each cell type. **(B - D)** Heatmaps of transcripts per million (TPM) from bulk RNA sequencing data demonstrating the expression of cell type-specific markers in control hiPSCs, nephron progenitor cells and hiPSC-Podocytes. Two different clones were analyzed for each cell type. Expression of **(B)** pluripotency, **(C)** nephron progenitor cells-related, and **(D)** podocyte-associated marker genes. **(E)** Validation of cell type-specific marker expression during the differentiation process was analyzed by qPCR in dermal fibroblasts, hiPSCs, nephron progenitor cells and terminal differentiated hiPSC-Podocytes. The fibroblast marker *ACTA* is upregulated in dermal fibroblasts, pluripotency marker *NANOG*, *POU5F1*, *SOX2* and *REX1* are expressed in hiPSCs only decrease and podocyte-specific marker *SYNPO* and *MAFB* are upregulated after initiation of differentiation. One-way ANOVA, ** p<0.01, *** p<0.001, **** p<0.0001.


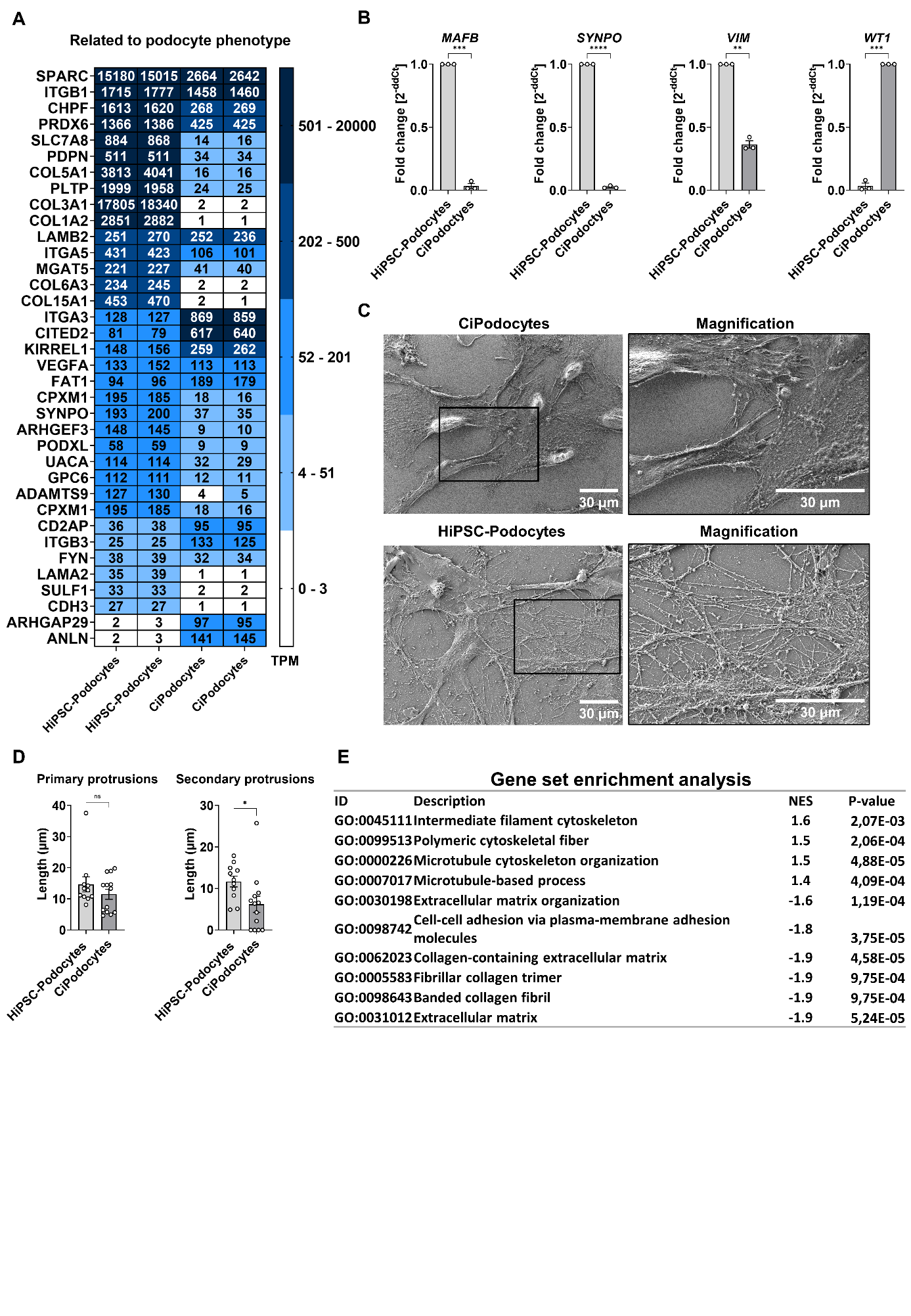


Figure S2. **Generated hiPSC-Podocytes derived from a healthy control donor in 2D culture can be defined as podocytes with a more advanced phenotype than ciPodocytes. (A)** Transcripts per million (TPM) from bulk RNA sequencing analysis of many genes associated with the podocyte cell type are displayed in a heatmap. Two different clones were analyzed for each cell line. **(B)** Fold change of mRNA levels of the podocyte-related markers *SYNPO*, *MAFB*, *VIM* and *WT1*. Data was normalized to *ACTB*. Welch’s t-test, ** p<0.01, p ***<0.001, **** p<0.0001. **(C)** Scanning electron microscope images show ultrastructural differences in cell morphology. Scale bars represent 30 µm. **(D)** Quantification of the mean length of primary and secondary protrusions given as length in µm. Welch’s t-test, ns: non-significant, * p<0.05. **(E)** Gene set enrichment analysis of differentially expressed genes. A positive score indicates pathways that are more active in hiPSC-Podocytes, while a negative score indicates pathways that are more active in ciPodocytes.


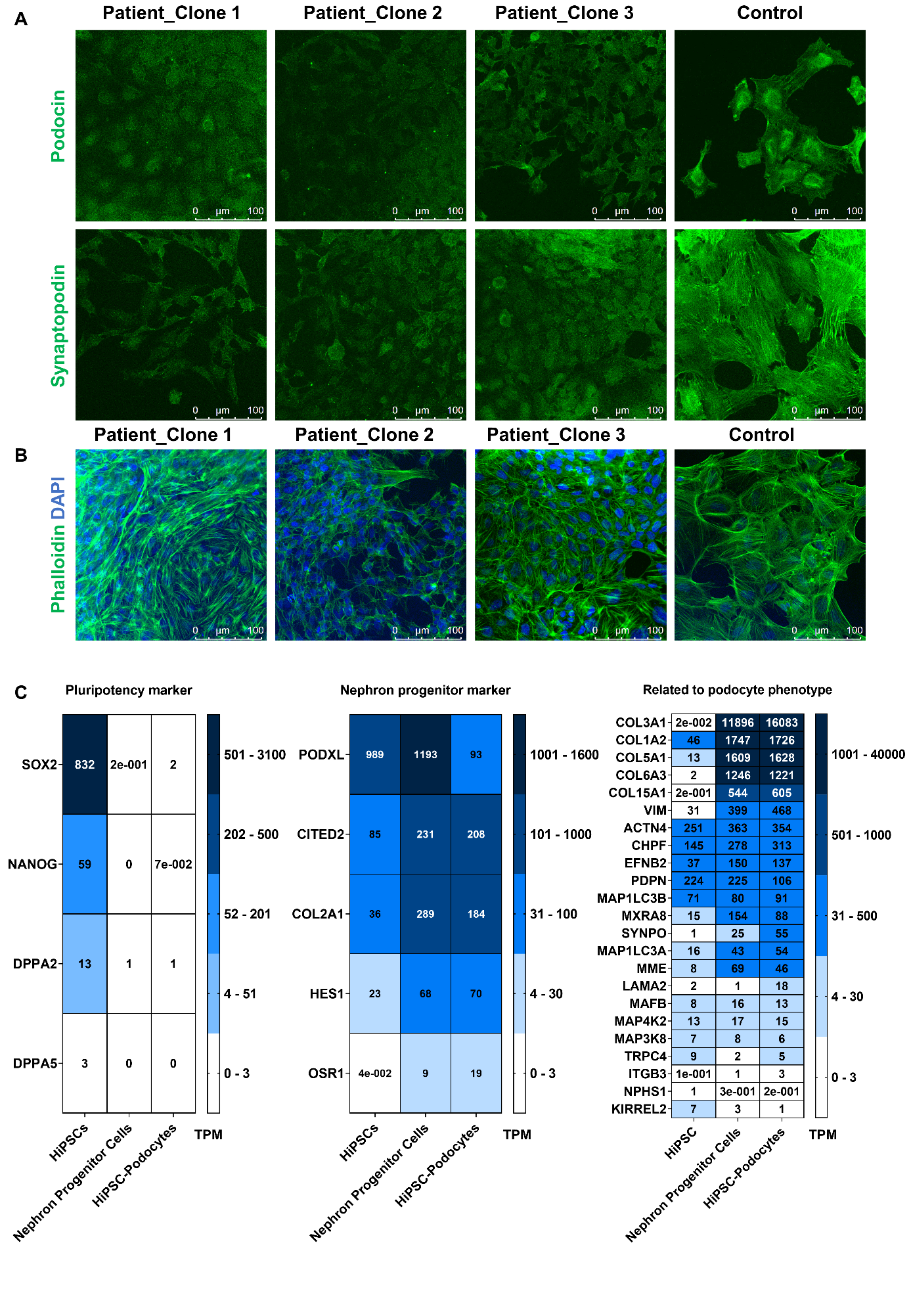


Figure S3. **Differentiation efficiency of patient-specific hiPSC-Podocytes derived from three individual clones. (A)** Podocyte marker proteins, such as podocin and synaptopodin, are expressed in all patient-specific clones. **(B)** Phalloidin (green) co-stained with DAPI (blue) to display the ability to form monolayers. Scale bars represent 100 µm. **(C)** Heatmaps of transcripts per million (TPM) from bulk RNA sequencing analysis showing the expression of cell type-specific markers in patient-specific hiPSCs, nephron progenitor cells and hiPSC-Podocytes. Two different clones were analyzed for each cell type.


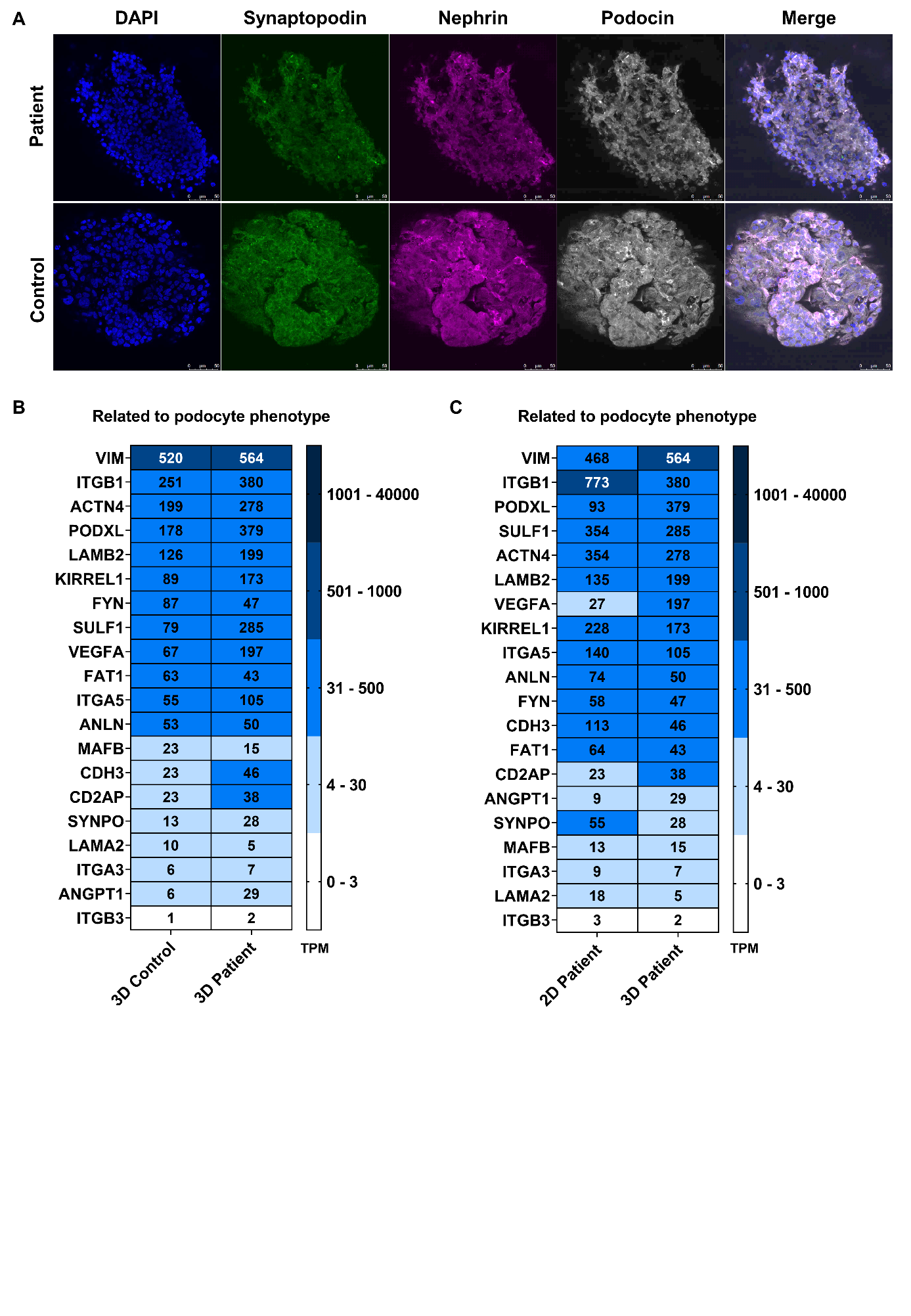


Figure S4. **Patient-specific hiPSC-Podocyte differentiated in a 3D microenvironment exhibit podocyte marker. (A)** Immunofluorescent staining of the podocyte marker proteins synaptopodin (green), nephrin (magenta) and podocin (grey). The 3D cultures were derived from embryoid bodies formed from patient and control hiPSCs, following the same differentiation protocol used for 2D hiPSC-Podocytes at 60 rpm on a shaking platform. Scale bars represent 50 µm. **(B - C)** Transcripts per million (TPM) from bulk RNA sequencing analysis of genes related to podocyte phenotype are displayed as the mean. Here, control- and patient-specific 3D hiPSC-Podocyte cultures **(B)** or rather patient-specific hiPSC-Podocyte cultures differentiated in 2D and 3D cultures **(C)** were compared.


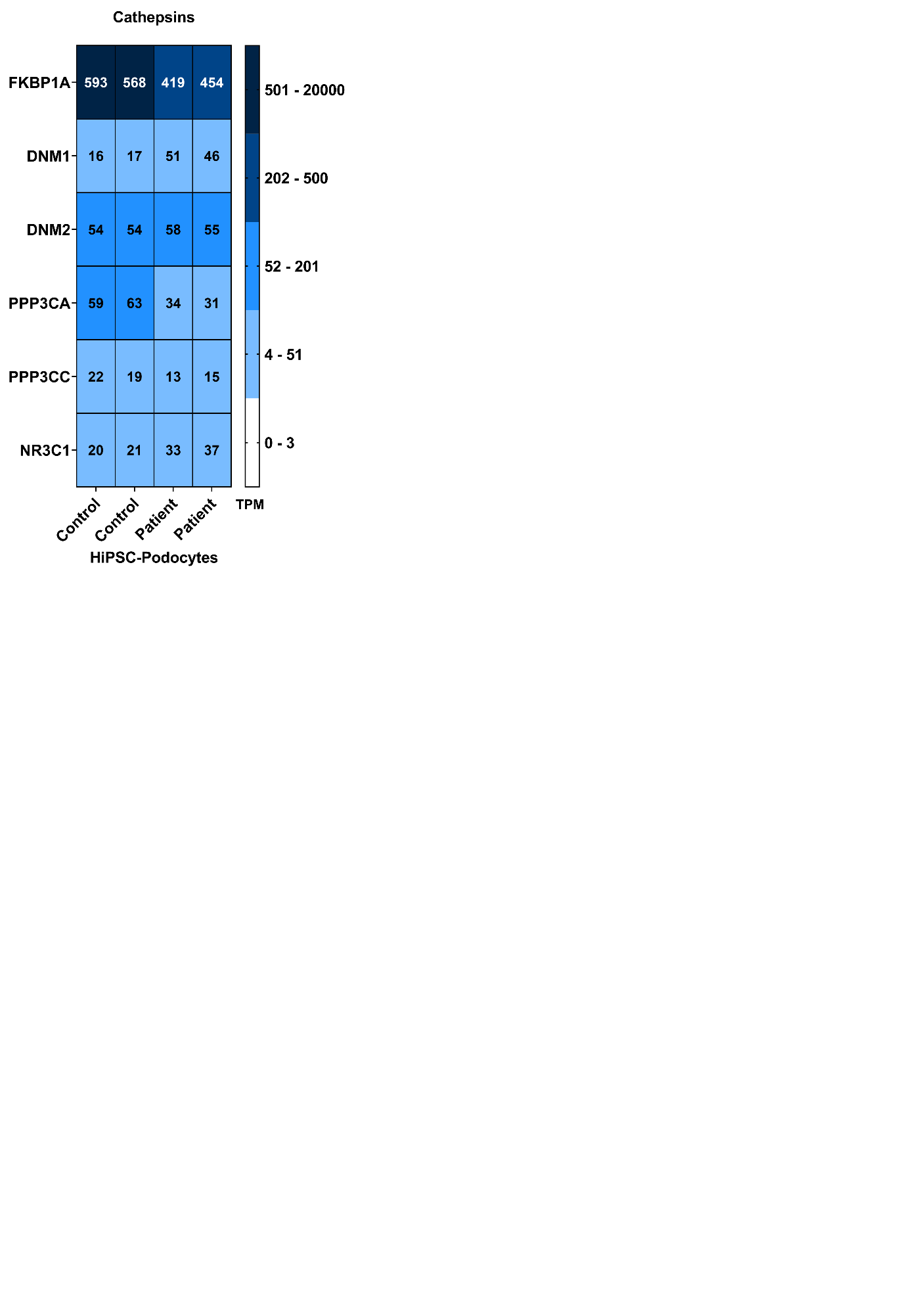


Figure S5. **Transcriptomic expression of cathepsin isoforms in control and patient hiPSC-Podocytes from bulk RNA sequencing data.** Transcripts per million (TPM) were compared for the cathepsin isoforms L, B, D, Z, A, C, V, K, O, and H. While cathepsins L, B, D, Z, and A were less expressed in patient hiPSC-Podocytes, cathepsin C and V were higher expressed compared to control hiPSC-Podocytes. Bulk RNA sequencing was performed of two individual clones for each cell line.


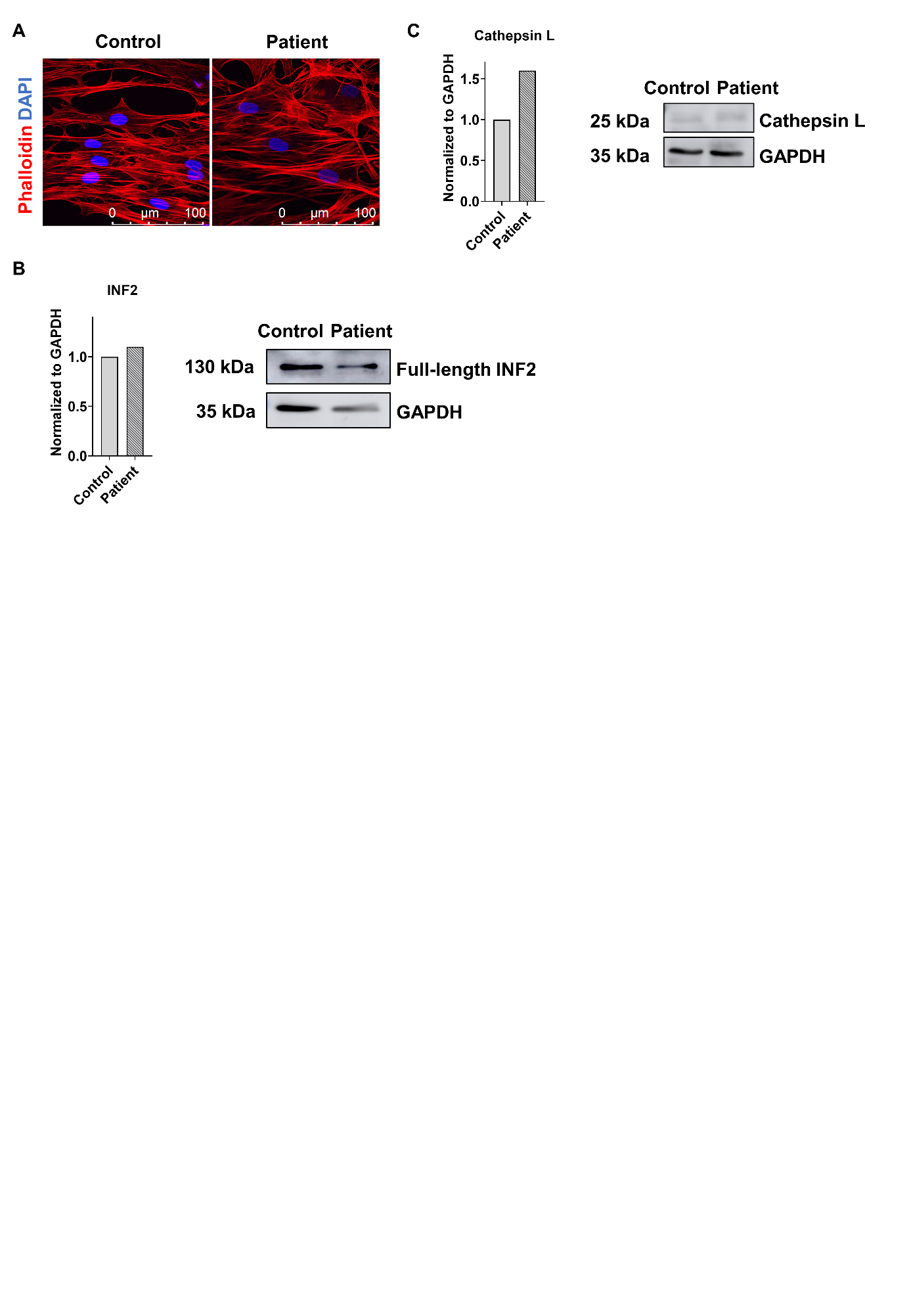


Figure S6. **Healthy control- and patient-derived dermal fibroblast display no alterations of the actin filaments, INF2 and cathepsin L protein expression.** **(A)** Phalloidin (red) and DAPI (blue) staining of dermal fibroblasts showing an unaltered actin cytoskeleton in both cell lines. Scale bars represent 100 µm. **(B)** INF2 and **(C)** cathepsin L protein levels were analyzed by Western blot analysis and normalization to GAPDH. n=1.


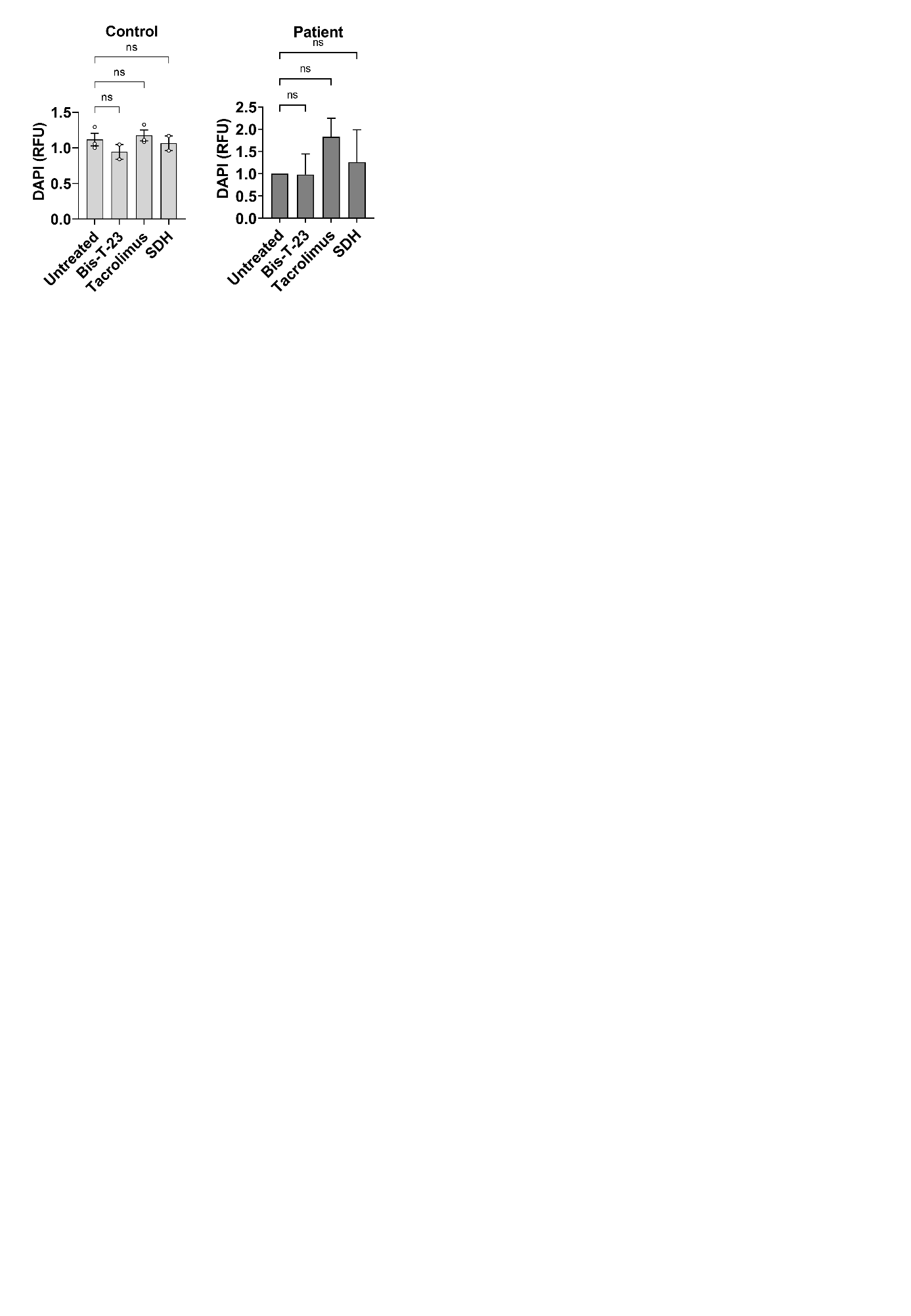


Figure S7. **Equal cell number after treatment experiments visualized by quantified DAPI signal.** Quantification of relative fluorescence units (RFU) of DAPI in control and patient hiPSC-Podocytes, both untreated and after 1 hour of treatment with Bis-T-23 (30 µM), tacrolimus (10 µg/mL), and SDH (1 µg/mL) in 96-well format. This analysis was performed in triplicate for each of the three clones using a plate reader. One-way ANOVA showing no significant differences, n=3.
